# Integrating protein structures and precomputed genealogies in the Magnum database: Examples with cellular retinoid binding proteins

**DOI:** 10.1186/1471-2105-7-89

**Published:** 2006-02-23

**Authors:** Michael E Bradley, Steven A Benner

**Affiliations:** 1 Department of Chemistry, University of Florida, P.O. Box 117200, Gainesville, FL, 32611, USA; 2 Foundation for Applied Molecular Evolution, 1115 NW 14^th ^Avenue, Gainesville, FL, 32601, USA; 3 Division of Biological Sciences, Department of Ecology and Evolution, University of Chicago, 1101 East 57^th ^Street, Chicago, IL, 60615, USA

## Abstract

**Background:**

When accurate models for the divergent evolution of protein sequences are integrated with complementary biological information, such as folded protein structures, analyses of the combined data often lead to new hypotheses about molecular physiology. This represents an excellent example of how bioinformatics can be used to guide experimental research. However, progress in this direction has been slowed by the lack of a publicly available resource suitable for general use.

**Results:**

The precomputed Magnum database offers a solution to this problem for ca. 1,800 full-length protein families with at least one crystal structure. The Magnum deliverables include 1) multiple sequence alignments, 2) mapping of alignment sites to crystal structure sites, 3) phylogenetic trees, 4) inferred ancestral sequences at internal tree nodes, and 5) amino acid replacements along tree branches. Comprehensive evaluations revealed that the automated procedures used to construct Magnum produced accurate models of how proteins divergently evolve, or genealogies, and correctly integrated these with the structural data. To demonstrate Magnum's capabilities, we asked for amino acid replacements requiring three nucleotide substitutions, located at internal protein structure sites, and occurring on short phylogenetic tree branches. In the cellular retinoid binding protein family a site that potentially modulates ligand binding affinity was discovered. Recruitment of cellular retinol binding protein to function as a lens crystallin in the diurnal gecko afforded another opportunity to showcase the predictive value of a browsable database containing branch replacement patterns integrated with protein structures.

**Conclusion:**

We integrated two areas of protein science, evolution and structure, on a large scale and created a precomputed database, known as Magnum, which is the first freely available resource of its kind. Magnum provides evolutionary and structural bioinformatics resources that are useful for identifying experimentally testable hypotheses about the molecular basis of protein behaviors and functions, as illustrated with the examples from the cellular retinoid binding proteins.

## Background

The amino acid sequences from a set of homologous proteins contain an imperfect record of the history of sequence divergence within that protein family. Much of this history can be modeled by a process that formally reconstructs the sequences of ancestral proteins throughout an evolutionary tree, given a multiple sequence alignment relating individual sites in the descendent proteins [1]. These reconstructions are generally presented in probabilistic form, where the likelihood that each of the standard amino acids occupied a particular site at a point in the tree is represented by a vector whose coefficients sum to unity, and where each coefficient represents the probability, conditional on the reconstruction model, that each of the standard amino acids occupied that site at that point.

Reconstructed ancestral sequences can be viewed as an expedient representation of extant sequence data, because they include all of the sequence information in a way that represents a best guess model for the historical reality. It is not surprising, therefore, that explicitly considering reconstructed ancestral sequences is a powerful tool for interpreting sequence data. For example, explicit consideration of ancestral states provided an understanding of the general features of correlated changes in proteins [2], an understanding that eluded a perplexed literature that attempted to analyze correlated change by leaf-leaf comparisons [3-6]. Likewise, many compelling tools for predicting the folded structure of proteins are based on analyses that consider reconstructed ancestral sequences [7,8]. Ancestral sequence reconstructions have provided insight as well into the relation between protein sequence, behavior, and adaptive function in proteins as diverse as ribonucleotide reductase [9], leptin [10], aromatase [11], sulfotransferase [12], steroid receptor [13], alcohol dehydrogenase [14], elongation factor [15], ribonuclease and its homologs [16,17], rhodopsin [18], lysozyme [19], and biofluorescent proteins [20]. In the last seven cases, ancestral proteins from extinct organisms were actually resurrected in the laboratory (reviewed in [21]).

As valuable as such studies are, however, only a very few scientists pursue them. Two reasons explain, at least in part, why studies involving reconstructed ancestral proteins are challenging. First, evolutionary analyses are highly dependent on the availability of a large amount of data collected by others. An evolutionary analysis of a protein family can generate interesting biological interpretations only if it contains a sizeable number of members, and only if those sequences are contributed from interesting organisms. Further, the analysis depends on the extent to which the sequences have diverged, and how the tree interconnecting the sequences is articulated.

Non-sequence information to support an evolutionary analysis of sequence data is also usually available only opportunistically. Thus, the availability of a crystal structure for a member of a protein family is generally not determined by a desire to support an evolutionary analysis. Likewise, there is no guarantee that a fossil record will exist for organisms holding ancestral proteins of interest, or kinetic data will be collected on the interesting proteins as biomolecules, or even that an interesting physiological recruitment has been studied by cell or organismic biologists.

This contingency means that one rarely knows in advance when one sets out to perform an evolutionary analysis what will be discovered. Generally the most interesting insights are true discoveries, coming opportunistically by those who can resourcefully take advantage of all information, especially that from researchers who delivered it to the literature for reasons quite different from the desire to support an evolutionary analysis.

This places a high value on database architectures that support browsing. The most useful database for an evolutionary bioinformaticist must allow the user to ask "What if?" and "Why not?" questions about the evolutionary histories of multiple families, where the answers can be extracted from the database in minutes or hours, not days or weeks.

This generates the second reason why detailed evolutionary bioinformatic analyses are rarely done. The public databases are inadequate for those who wish to browse, especially if they hope to collect information about ancestral protein sequences. The decision to do an evolutionary analysis with the NCBI web page is a decision to spend weeks doing expert interpretation of BLAST outputs, multiple sequence alignments, and tree constructs. This means that evolutionary analyses are today done one family at a time, by experts in evolutionary analysis, and are not done by bench experimental scientists who have the most access to the non-sequence information that enriches a purely bioinformatics study.

A naturally organized commercial database incorporating information from ancestral sequences, known as the MasterCatalog, was introduced in 2000 to support browsing [22]. It has been remarkably successful doing so in the hands of those who have had access to it [10-12,23,24]. The product falls between market niches, however; too expensive to be available to academic scientists, yet generating too little revenue to attract major capital support. Therefore, it has not permitted broad based discovery using genomic sequence data. Some time ago, we set out to develop a publicly available database that would support browsing of evolutionary and structural data jointly. We have been able here to assemble such a database, Magnum, that covers protein families where at least one member has a crystal structure.

We report here a characterization of the Magnum database, which covers the multiple sequence alignments, phylogenetic trees, inferred ancestral sequences, amino acid residue replacements on branches, and crystal structures. We demonstrate the utility of Magnum as a research tool for addressing general trends in protein evolution as well as more specific questions of relevance to individual protein families. In this case, we performed queries to select individual amino acid replacements occurring along short branches, located at internally folded protein sites, and requiring a nucleotide change at each of the three codon positions for interconversion. The results lead us to, among others, the family of proteins that bind retinol and other hydrophobic ligands, where we discovered along one branch a replacement that may explain physiological differences in ligand binding specificities, and along another branch a set of replacements related to the recruitment of this protein as an eye lens crystallin in diurnal geckos.

## Results

### Description of magnum

**Figure 1:**
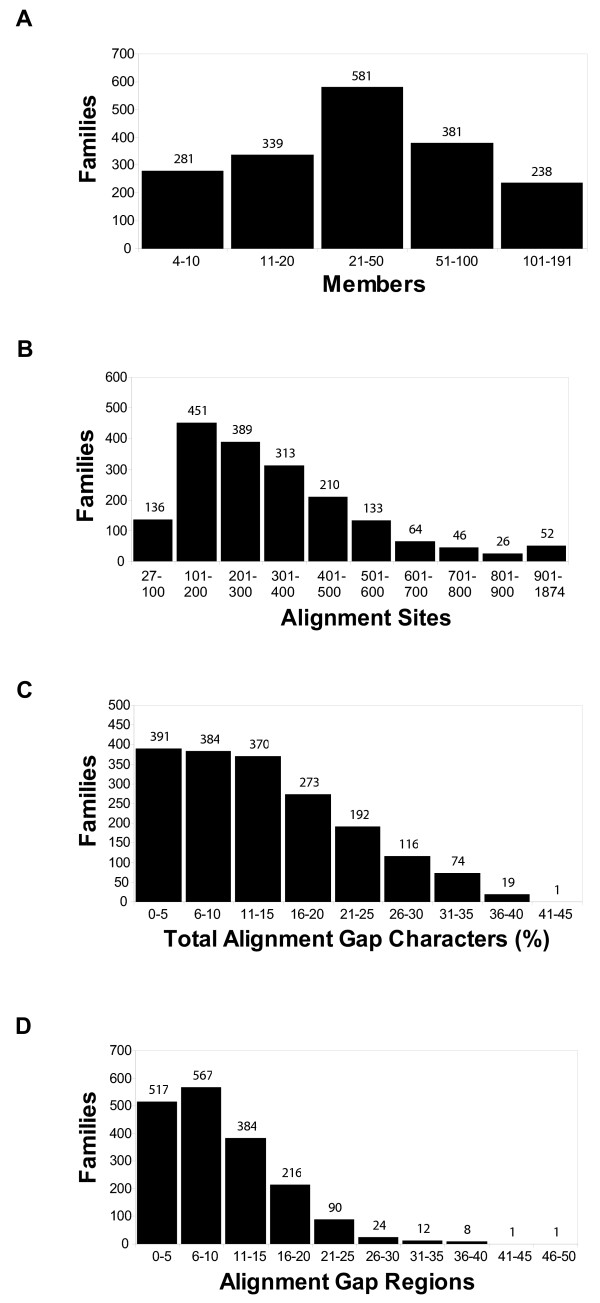
Alignments. Histograms showing the distribution of protein sequence families (y-axis, number of families) in Magnum versus (A) Number of homologous family members, (B) Number of aligned sites in the multiple sequence alignment of the family, (C) Percentage of characters that are gaps in the multiple sequence alignment, and (D) Number of regions containing gaps.

In its current form, Magnum comprises 1,820 "Superfamilies" [25] from the Protein Information Resource (PIR) containing from 4 to 191 family members. Together, these contain a total of 85,386 protein sequences (Figure 1A). The average family has 43 sequences aligned over 333 sites (Figure 1B). If all of the alignments were concatenated, the total alignment length would be 606,638 sites.

Of the 606,638 alignment sites, 68% were completely gap-free. Where gaps do occur, they comprise less than 15% of all alignments characters for approximately two-thirds of the families (Figure 1C) and are concentrated within ten or fewer distinct regions for the typical family (Figure 1D).

**Figure 2:**
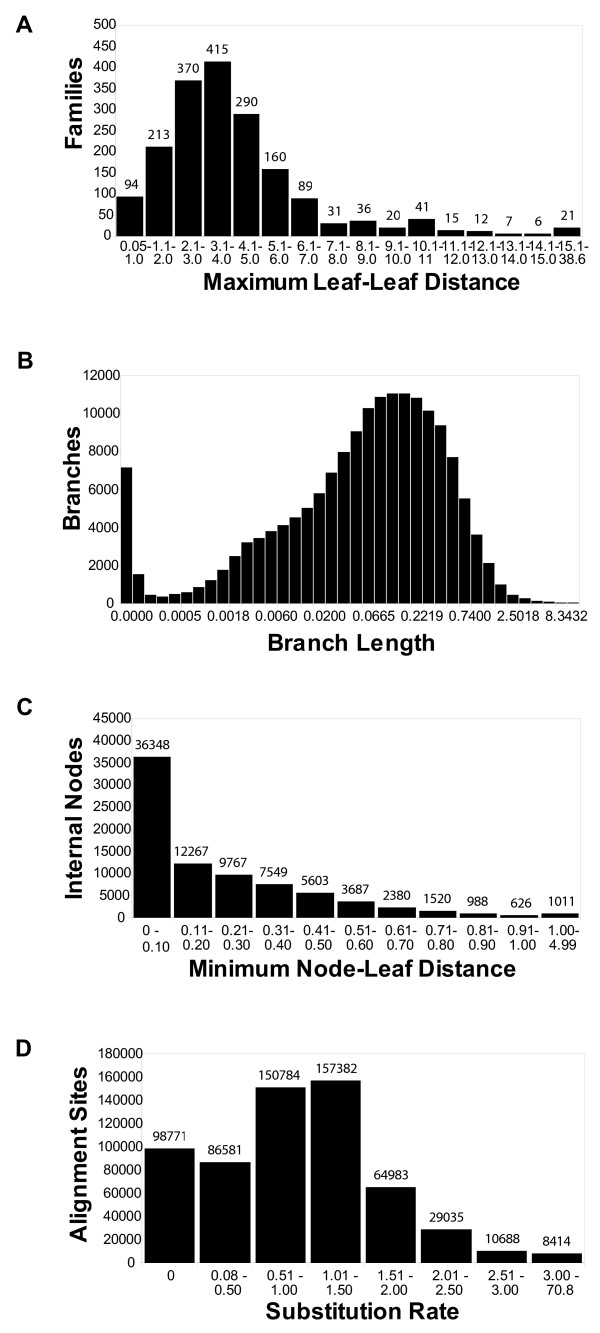
Phylogenies. Histograms showing the distribution of families, branches, nodes, and sites as a function of the evolutionary feature indicated (x-axis). (A) Width of the evolutionary tree interconnecting the family members, (B) Length of the tree branches, (C) Shortest distance from internal nodes to a leaf node, (D) Substitution rate factor. (A-C) Units are replacements per site.

The average family in Magnum has a width of 4.2 amino acid replacements per site (Figure 2A). The average branch represents a distance of 0.2 replacements per site (Figure 2B). An unrooted phylogeny with *n *leaf sequences has *n*-2 internal nodes and 2*n*-3 branches. The trees collected in Magnum contain 81,746 internal nodes and 165,312 branches. Over 80% of internal nodes are less than 0.4 amino acid substitutions per site distant from a leaf sequence (Figure 2C). A substantial fraction of these connected an internal node to a leaf. The remaining 20% of the nodes, nearly all not directly connected to a leaf, were more than 0.4 amino acid substitutions per site distant from a leaf. The distribution of substitution rates at individual sites is shown in Figure 2D.

### Accuracy and error in reconstructed sequences

To obtain a general measure of the reconstruction accuracy at the typical site in the Magnum database, conditional posterior probabilities were obtained from the marginal reconstruction method, which independently treats each site at each internal node in order to calculate the probability of deriving the leaf sequences starting from each of the 20 amino acids at that position in the tree (see Methods). These probabilities are conditional on the parameters of the metatheory used during the reconstruction. As the accuracy of a reconstruction declines, so to does the posterior probability value generated by the scoring tool.

In Magnum, 31,283,104 ancestral sites were reconstructed. At 6,022,509 sites, the reconstruction suggested gap characters, which are not assigned a probability value. Although we do not consider replacements involving gaps in this study, we chose to model the gapping history because even a simple representation of the indel process was preferable to ignoring it. Where the reconstructions did not contain gaps, the overwhelming majority of conditional posterior probabilities for the primary amino acids (the preferred amino acid reconstructed at a site in a nodal sequence) were above 90% (Table 1).

**Table 1:** Marginal versus joint reconstruction

posterior probability	joint = marginal _1_	joint ≠ marginal _1_			
		
		marginal _2_	marginal _3_	marginal _4_	marginal _5–20_
1 – 10%	1,238	181	122	112	401
11 – 20%	75,322	16,354	10,065	6,285	11,801
21 – 30%	261,119	66,689	31,945	13,920	11,556
31 – 40%	462,681	116,133	37,273	8,923	4,508
41 – 50%	645,994	146,765	19,181	3,320	1,587
51 – 60%	823,937	146,931	7,127	1,207	522
61 – 70%	759,976	57,359	2,576	414	158
71 – 80%	789,136	18,078	799	124	66
81 – 90%	1,007,528	4,057	215	30	5
91 – 100%	20,433,891	431	24	3	3
	25,260,822	572,978	109,327	34,338	30,607

All reconstructed sites are cross-tabulated according to the posterior probability of the best marginal reconstruction amino acid and the marginal reconstruction rank, from highest to lowest posterior probability, of the sole joint reconstruction amino acid.

The primary amino acid residues reconstructed using marginal and joint methods were then compared. Discrepancies between these reconstructions would indicate disagreement, which would imply uncertainty in reconstructions arising from an irresolvable concern over which of the two methods is more likely to capture the historical reality. Fortunately, the primary residue inferred using the marginal reconstruction method differed from that inferred by the joint reconstruction method at only 2.8% of the ungapped ancestral sites (747,557 sites, see Table 1). These sites predominantly occurred among sites where the marginal posterior probabilities were low. This indicates that disagreement between the "marginal reconstruction" and "joint reconstruction" metatheories is found primarily at sites where the inferences of the ancestral amino acid are not confident in any case. At the majority of these sites, the residue inferred by the joint reconstruction method was the second or the third most likely residue inferred by the marginal reconstruction method.

From these results, we conclude that the majority of ancestral reconstructions in Magnum are fairly accurate and robust to the reconstruction philosophy (joint versus marginal). More precisely, inferences drawn directly from the Magnum database without further refinement of its ancestral characters are not likely to be dominated by database error. Further, when performing detailed analyses with the data, numerous indicators of accuracy are available from Magnum to the user for deciding where the reconstructions are likely to be less certain.

### Structural characteristics of the families

**Figure 3:**
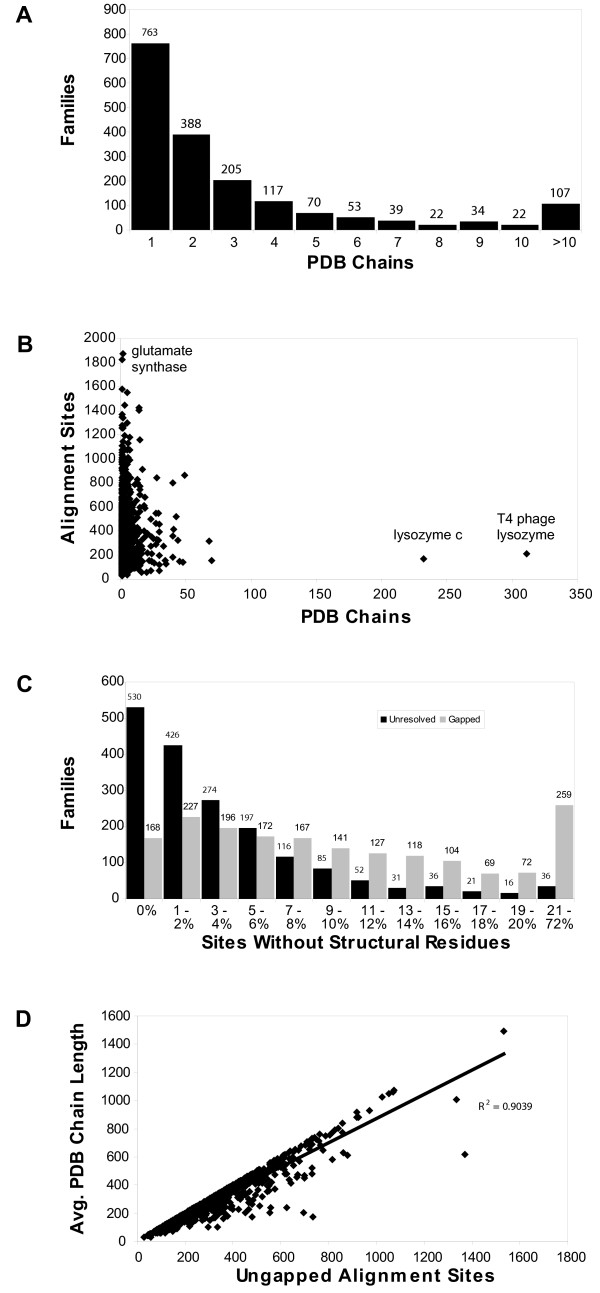
Structures. (A) Histogram showing the distribution of PDB chains associated with individual families in Magnum. (B) Scatter plot of alignment length versus the number of PDB chains for each family. Glutamate synthase has the longest alignment length; lysozyme c and T4 phage lysozyme have the largest number of non-redundant PDB chains. (C) Frequency histograms for the proportions of alignment sites without structural information due to indels (gray), and unresolved areas of the structure (black). (D) Average PDB chain length plotted against the number of sites aligned to at least one PDB chain residue for families with at least two PDB chains (the coefficient of determination calculated by linear regression is also shown).

As a condition for inclusion within Magnum, each PIR Superfamily had to be associated with at least one protein chain from a structure in the Protein Data Bank (PDB). The median Magnum family has members associated with two PDB chains (Figure 3A). The families with the longest alignments have the fewest PDB chains and vice versa (Figure 3B). We explain this by the observation that short sequences are more prevalent in the PDB.

Multiple sequence alignment was used to relate PDB chain sites with family member sites. At 80,373 of the final sites, gaps were placed within the PDB chain sequences (Figure 3C). At an additional 22,571 sites, residues in the PDB-listed sequence were aligned, but the PDB database did not report resolved crystallographic coordinates for their side chains, and therefore secondary structural information was unavailable (Figure 3C). After correcting for gaps and unresolved residues, 503,694 sites were aligned with at least one resolved residue from the PDB.

In the current version of Magnum, 1,057 families are associated with multiple PDB chains (Figure 3A). For the majority of these families, a strong correlation exists between the average length of the PDB chains and the alignment length after correcting for sites without structural information (Figure 3D). This demonstrates that in many families with multiple structures the same alignment sites are covered by each PDB chain.

The structural information was then used to assign surface accessibility and secondary structural characteristics for the 503,694 sites where it was available (Table 2). If more than one PDB chain was applicable to a site, the side chain accessibility values were averaged. At the 426 and 6,416 sites where four and three, respectively, secondary structure types were assigned we report the most common type. In the event that none of the secondary structures are more common than the others, such as in the 56,877 sites with exactly two assignments, the secondary structure is reported according to the following hierarchy: turn, helix, coil, and then strand.

**Table 2:** Surface accessibility and secondary structure

surface accessibility*	secondary structure**				
	
	helix	strand	coil	turn	total
1 – 10%	60,833	48,079	23,319	19,417	151,648
11 – 20%	16,411	9,849	11,260	10,601	48,121
21 – 30%	13,424	7,374	9,147	9,695	39,640
31 – 40%	12,014	6,158	7,857	9,869	35,898
41 – 50%	10,923	4,992	7,131	9,748	32,794
51 – 60%	10,578	4,372	6,484	9,575	31,009
61 – 70%	9,856	3,745	5,825	9,465	28,891
71 – 80%	9,130	3,141	5,149	8,207	25,627
81 – 90%	8,232	2,583	4,250	7,470	22,535
91 – 100%	30,045	6,573	17,945	32,968	87,531
	181,446	96,866	98,367	127,015	503,694

* The average surface accessibility is reported when multiple PDB chains are aligned at a site.

** The most commonly called secondary structure type is reported when multiple PDB chains are aligned at a site.

Residues at approximately half of the sites were less than 30% accessible. This is a threshold at which we distinguish 'buried' and 'exposed' sites for the studies below, although the user is able to select alternative cutoffs. The four secondary structure types were each well represented at all levels of surface accessibility. Residues in strands were more likely to be buried, while residues in turns were more likely to be found on the surface [26]. Residues in helices and coils did not show a preference for buried or exposed sites.

### Amino acid residue replacements along branches

As a further test of the uncertainty in the ancestral sequence reconstructions, we asked whether patterns in the replacement of amino acids as represented by replacements inferred from ancestral sequences were similar to/different from those patterns obtained by many workers (e.g., Dayhoff [27]) by comparing extant sequences. For short, medium and long branches, 10,000 leaf-leaf and node-node pairs were randomly selected. Replacements were counted within these six collections of aligned sequences, and Dayhoff matrices were constructed from the replacement counts [see Additional file 1].

For the node-node comparisons, replacements were counted using three tools that differed in the weighting of replacements. The 'best count' tool assigned equal weight to all replacements, which were detected by searching for sites where the most probable parent residue differed from the most probable residue at the same site in the child nodal sequence. Using the same strategy to search for replacements, the 'best fractional' tool weights each replacement by the product of the marginal posterior probability values of the parent and child residues. The 'all fractional' tool exhaustively considers, for every aligned site, all possible combinations of parent and child residues weighted by their combined probability. In addition to the 190 pairs of different residues (i.e. replacement events), each tool also keeps a running total the 20 non-replacement pairs.

**Figure 4:**
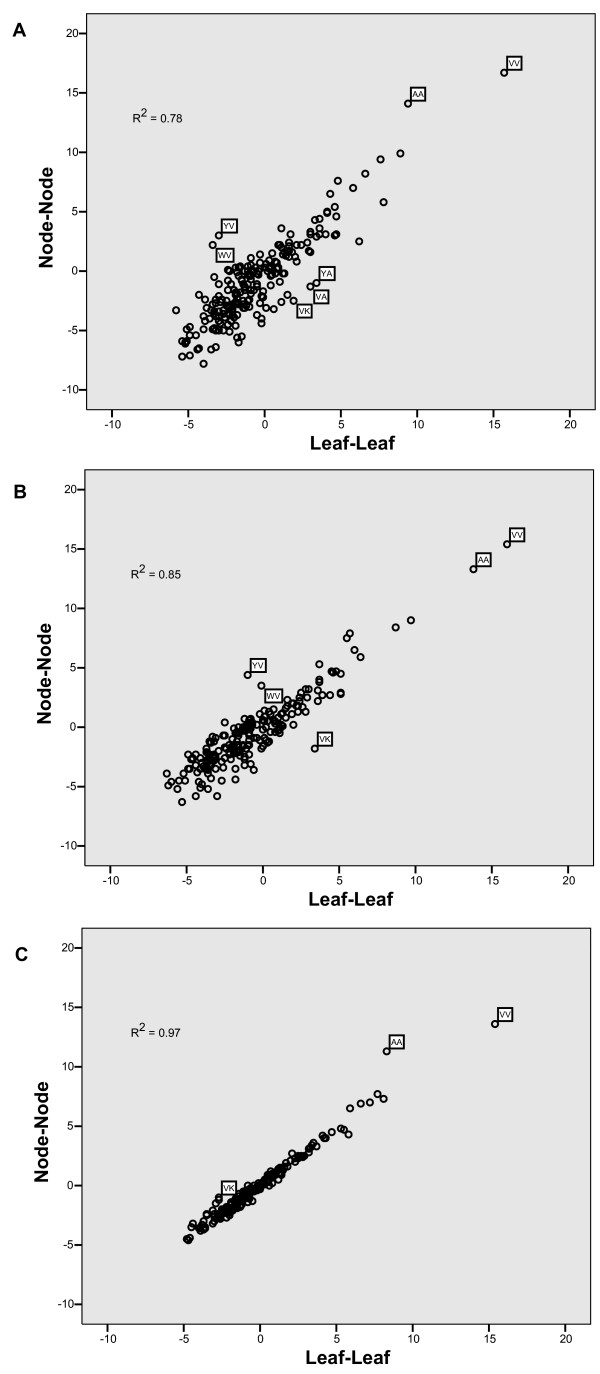
Dayhoff matrix comparisons. Elements of leaf-leaf (x-axis) and node-node (y-axis) Dayhoff matrices are plotted together from (A) short, (B) medium and (C) long branches. The coefficient of determination calculated by linear regression is shown. Labeled points involve the amino acids alanine or valine. The all fractional counting method was used in node-node comparisons shown. Similar results were obtained with the other counting methods (data not shown).

A clear linear relation was observed between corresponding node-node and leaf-leaf matrix elements at all three distances regardless of the replacement counting tool (Figure 4A–C and data not shown). From this, we conclude that replacement events reconstructed from node-node comparisons are similar in type to the amino acid replacements based on leaf-leaf comparisons.

Intriguingly, the correlation coefficient was larger in the long branches examined than in the medium and short branches. This phenomenon is not understood. Interestingly, the replacement pairs that deviate from the regression line in Fig. 4 appear most frequently to involve alanine or valine. Further, alanine and valine appear to be most conserved, regardless of the distance between the sequences. This is a discovery worth investigating in the future.

The branch lengths used in the Magnum trees are obtained by fitting the matrix of pairwise leaf-leaf distances (in units of replacements per site) upon the tree that gives the smallest total distance using the strategy in the program Weighbor [28]. Once the ancestral sequences are in hand, an alternative measure of the distance of a branch connecting two nodes (or a node-leaf branch) was obtained by examining two (or one) ancestral sequences, and counting the number of changes, weighted by the fractional probabilities of those residues where appropriate. These are "branch-lengths-from-reconstruction".

The number of changes on any branch at any individual site cannot exceed unity, as multiple substitutions at a site are not captured by simply comparing two sequences. Thus, the branch-lengths-from-reconstruction, a metric that averages these over all sites, cannot exceed unity. The branch-lengths-from-reconstruction with the all fractional tool closely resembled the actual branch lengths, especially at short distances (Table 3). The other tools rarely overestimated the actual branch length (Table 3). Where branch-lengths-from-reconstruction are less than actual lengths, one explanation is that intermediate replacements occurred at some sites. By correcting for multiple substitutions, which occur more frequently on longer branches and for sites with higher substitution rates, it would be possible to improve the correspondence between branch-lengths-from-reconstruction and the actual tree branch lengths.

**Table 3:** Branch-lengths-from-reconstruction versus Tree-branch-length

	Branch-lengths-from-reconstruction*	Tree-branch-length										
			
		0	.0001 – .006	.0061 – .020	.0201 – .040	.0401 – .070	.0701 – .130	.1301 – .220	.2201 – .400	.4001 – 1.00	≥ 1.001	
AF												
	0	**5,891**	1,292	6	4	0	0	0	0	0	3	7,196
	.0001 – .006	412	**16,365**	2,268	129	66	29	1	1	0	10	19,281
	.0061 – .020	130	1,576	**11,110**	1,829	100	93	47	1	2	12	14,900
	.0201 – .040	99	410	2,406	**7,070**	2,456	146	80	31	1	1	12,700
	.0401 – .070	145	274	1,591	2,795	**7,045**	3,475	125	66	5	5	15,526
	.0701 – .130	191	221	1,555	3,717	4,645	**11,268**	5,841	305	33	1	27,777
	.1301 – .220	169	89	474	1,673	3,396	5,753	**10,051**	10,022	583	4	32,214
	.2201 – .400	100	28	88	237	666	1,866	2,848	**8,811**	14,136	626	29,406
	.4001 – 1.00	10	1	2	3	8	48	63	178	**2,492**	3,507	6,312
BF												
	0	**7,131**	7,791	1,260	295	137	103	51	23	8	7	16,806
	.0001 – .006	11	**11,570**	4,748	1,282	575	275	90	28	15	13	18,607
	.0061 – .020	0	883	**12,873**	9,119	4,506	2,641	883	232	51	19	31,207
	.0201 – .040	0	10	606	**6,487**	8,980	5,554	2,046	779	175	13	24,650
	.0401 – .070	0	1	12	269	**4,067**	10,172	4,592	1,770	559	60	21,502
	.0701 – .130	1	0	0	5	115	**3,887**	10,244	8,278	2,757	252	25,539
	.1301 – .220	0	0	0	0	2	46	**1,142**	7,954	8,907	1,043	19,094
	.2201 – .400	4	0	0	0	0	0	7	**350**	4,737	2,175	7,273
	.4001 – 1.00	0	1	1	0	0	0	1	1	**43**	587	634
BC												
	0	**7,142**	17,555	2,581	405	173	128	71	30	10	13	28,108
	.0001 – .006	0	**0**	0	0	0	0	0	0	0	0	0
	.0061 – .020	0	2,683	**15,870**	7,557	1,317	340	122	59	21	13	27,982
	.0201 – .040	0	16	988	**8,382**	7,874	1,731	277	77	17	5	19,367
	.0401 – .070	0	1	60	1,074	**8,310**	9,621	1,240	217	46	6	20,575
	.0701 – .130	0	0	0	38	698	**10,587**	11,484	2,207	264	13	25,291
	.1301 – .220	0	0	0	1	10	270	**5,813**	13,177	2,598	36	21,905
	.2201 – .400	5	0	0	0	0	1	48	**3,646**	13,213	1,120	18,033
	.4001 – 1.00	0	1	1	0	0	0	1	2	**1,083**	2,963	4,051

*Replacements per site from all fractional (AF), best fractional (BF), and best count (BC) tools.

### A browsing example: F-to-E, F-to-Q, and F-to-K replacements

We illustrate an example of how the Magnum database can be used to ask a question about amino acid replacement in general, where possible answers to the question bear on evolutionary and structural biology.

**Figure 5:**
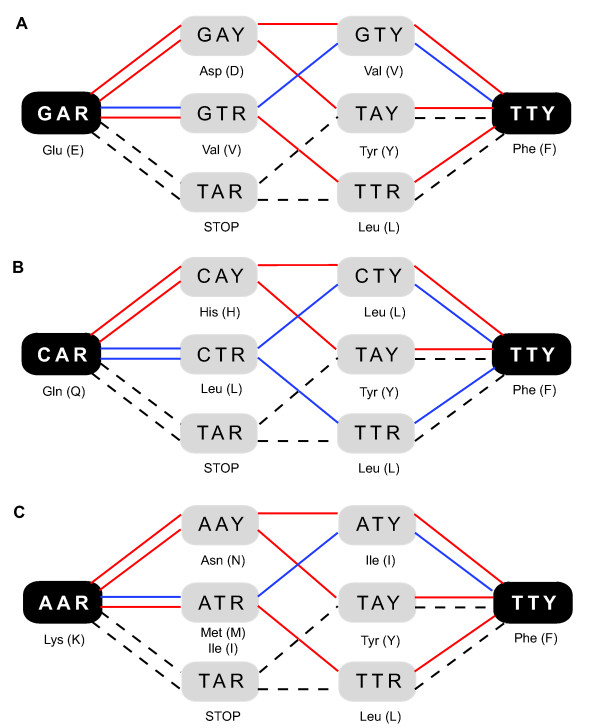
F-to-E/Q/K replacement pathways. Start and end residues are black with white codons, intermediate residues are gray with black codons. Codons are written using DNA symbols (A, adenine, T, thymine, C, cytosine, G, guanine, Y, pyrimidine, R, purine). Red pathways cross two different residues; blue pathways cross one residue; dashed pathways cross a stop codon. Note that dashed paths are identical in (A) F-to-E, (B) F-to-Q, and (C) F-to-K.

We started by noting, trivially, that 190 distinct amino acid replacement patterns exist given 20 standard amino acids and ignoring directionality in time (X-to-Y equals Y-to-X). In 14 of these, three nucleotide substitutions (one at each codon position) are required to effect the replacement, regardless of the codons used in the parent or child genes. Three of these pairs interconvert the hydrophobic amino acid phenylalanine (F) with one of the polar amino acids, lysine (K), glutamine (Q), or glutamate (E). For example, the F-to-K replacement requires the conversion of a TTY codon (where Y is a pyrimidine) to an AAR (where R is a purine), requiring a total of three transversions. The six shortest pathways for the F-to-E, F-to-Q, and F-to-K replacements are shown in Figure 5.

The F-to-E/Q/K replacements on branches of any length were found to be relatively infrequent, observed at only 0.026% of all recorded replacements (Table 4). The collection of F-to-E/Q/K replacements appears to be distributed with no obvious preference in large and small families, long or short alignments, or branches connecting sites with higher or lower posterior probability. The F-to-E/Q/K replacements are, however, found more frequently on longer branches and in sites holding side chains on the protein surface (Table 4). Each of these facts is readily retrieved given the structure of the Magnum database.

**Table 4:** F-to-E/Q/K replacements versus all 190 possible replacement patterns

	all pairs	F-to-E/Q/K	F-to-E	F-to-K	F-to-Q
replacements*	5,918,700	15,454	5,922	5,815	3,717
average probability**	0.641	0.567	0.563	0.564	0.577
distinct families	1,820	1,457	1,142	1,160	1,068
average members/family	46.9	54.1	60	60.8	62.5
distinct alignment sites	491,794	13,169	5,347	5,181	3,424
average substitution rate/site	1.12	1.48	1.44	1.49	1.52
sites with PDB data	442,102	12,101	4,932	4,734	3,172
protein surface sites	54%	72%	73%	74%	65%
distinct branches	148,522	11,800	5,235	5,089	3,456
average branch length	0.207	0.697	0.807	0.782	0.761

*Detected as sites where the most probable parent and child residues were different.

**The average product of probabilities for the parent and child residues.

In the hope of identifying F-to-E/Q/K replacements involved in adaptive change, buried sites on short branches (less than 0.12 substitutions per site) were selected. Again, the Magnum database permitted this information to be extracted with little effort. A total of 291 replacements satisfied these criteria [see Additional file 2].

First, the alignments and phylogenies were inspected to determine which of these events might reflect database error; misalignments were of special concern. Out of 41 events we found that 17 of the alignments were most likely incorrect. This is not surprising due to the unusual nature of the replacement events under inspection in this sample. We expect, therefore, that this is a worst case example.

### Cellular retinoid binding proteins

While each of the remaining F-to-E/Q/K replacements proved to be interesting, we choose to discuss here just one to illustrate how opportunities arise as one exploits a browsable database. In the example chosen for this illustration, an F-to-Q replacement was found at alignment site 7 in a family of beta barrel folded proteins that bind to assorted hydrophobic ligands, including sterols, fatty acids, and retinoids [see Additional file 3].

**Figure 6:**
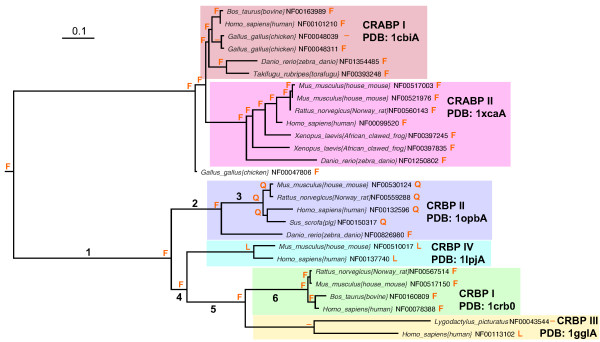
Phylogeny of retinoid binding proteins. Cellular retinoic acid binding protein (CRABP) subfamilies I and II and cellular retinol binding protein (CRBP) subfamilies I – IV are boxed. Extant and ancestral amino acids corresponding to alignment site 7 (PDB:1opbA position 4) are shown at leaf and internal nodes.

This particular F-to-Q replacement was found in a lineage leading to a subfamily of proteins, known as cellular retinol-binding protein II, after the mammals holding these proteins diverged from bony fishes (Figure 6; branch 3). A crystal structure of a member of this subfamily (135 amino acids, PDB: 1opbA) was used as a reference structure; the F-to-Q replacement occurs at position 4 in this protein.

We considered two hypotheses. The first, actually intimated in the literature [29,30], is that the residue at alignment site 7 influences the geometry of the glutamine at alignment site 122 (PDB:1opbA position 108) via hydrogen bonding. The glutamine at site 122 directly contacts the hydroxyl group of retinol, and the carbonyl group of retinal [29,30]. According to this model, F in site 7 forms a "pi anion" interaction with the amide hydrogen of site 122 glutamine, constraining it to offer its amide oxygen to either retinol or retinal, and therefore causing the protein to favor the first as a ligand over the second. A Q at site 7 would be able to hydrogen bond with either the amide oxygen or the amide hydrogen from Gln 122, allowing it (in turn) to offer either the amide hydrogen or the amide oxygen to the retinal or retinol (respectively), allowing it to bind either (but see [31]). The intermediary amino acids along the shortest pathways between F and Q do not each possess the chemical properties to perform this molecular physiological function. Leu cannot form a hydrogen bond of any kind. His can form hydrogen bonds that are analogous, although not geometrically identical, to those that can be formed by Phe or Gln. Tyr, in contrast, can form hydrogen bonds using the pi anion mechanism; indeed these are at least as strong as those formed by Phe.

The second hypothesis is that at least some of the intermediates between Phe and Gln are acceptable to the protein. Under this hypothesis additional sequence data that articulated the branch, sequences that were not available when version 2.62 of iProClass was established, might show that the intermediate steps were found.

The second proved to be the case. Motivated by the peculiarity of the F-to-Q event in this protein family, we searched for and found additional sequences from *Gallus gallus *(Genbank: XP422636) and *Xenopus tropicalis *(Genbank: DN068529). Further, both contained a Tyr at site 7. Therefore, articulating the tree along branch 3, we reconstructed a Tyr as an intermediate between the parent Phe and the derived Gln. This is, perhaps not accidentally, exactly the residue that would be chosen based on the chemical logic in the preceding paragraphs.

It is still necessary, of course, to take two steps to get from Tyr to Gln. Leu would presumably be unacceptable in this role; His might be tolerated. Although efforts to further articulate the tree must involve the sequencing of marsupials (these are in progress), we would predict that if the tree were articulated and an intermediate amino acid were detected, it would be His, not Leu.

Curiously, although Leu is not known in this lineage, it is known elsewhere among the cellular retinol binding protein paralogs. This fact is easily discoverable with Magnum, given its support of browsing. It is found in site 7 in several of the cousin families, including the cellular retinol-binding protein III, known in human and gecko (as a crystallin [32]), and cellular retinol-binding protein IV, known in mouse and human. Interestingly, the affinity of these for retinal and retinol was both very low: K_*d *_values of 60 and 200 nM [33,34], compared with 0.1 nM for the homologs that retain the ancestral F, and 10 nM for those with the derived Gln at site 7 [35,36].

**Figure 7:**
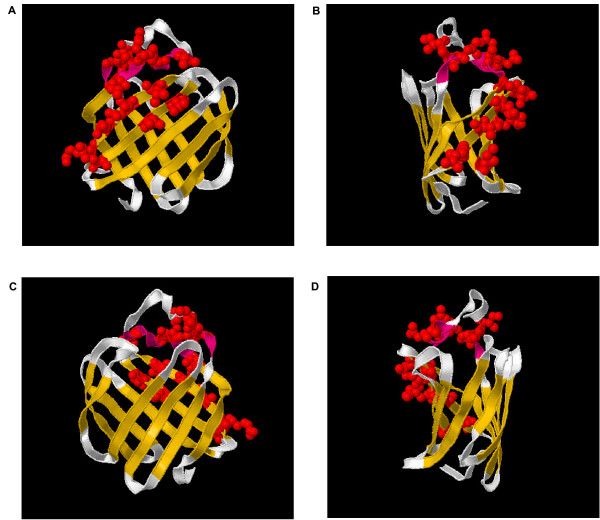
Gecko crystallin contact site prediction. 15 of the 25 sites experiencing amino acid replacement along the branch leading to the gecko crystallin protein (Figure 6; branch 7) are highlighted in red. The reference structure is PDB:1gglA. (A-D) Successive quarter turns of the molecule about its y-axis.

The opportunistic nature of database browsing, and the ability of the Magnum database to support it, was then illustrated again. The amino acid replacements along the branch leading to the gecko crystallin (Figure 4; branch 7) were delivered from the precomputed Magnum database [see Additional file 4], as was their distribution in the crystal structure of the nearest homolog (PDB:1gglA). Remarkably, the sites undergoing replacement along this branch were not randomly distributed. Rather, many of them were concentrated in strands I and J, and helices 1 and 2, on the amino acids presented to the surface, creating a highly modified surface (Figure 7). We expect that these are contact sites required for the formation of lens crystals. A planetary biology explanation as to why this protein, which binds the light absorber 3,4-didehydroretinol, evolved as geckos moved from a nocturnal lifestyle to a diurnal lifestyle is found in the literature [37].

## Discussion

The Magnum database is presented here as a tool to permit the rapid exploration of hypotheses relating to protein structure and function, using evolutionary analyses, and to support the pursuit of leads obtained by browsing. As required to obtain the full power of a combined evolutionary and structural analysis, Magnum integrates crystallographic protein data with detailed evolutionary features, such as amino acid replacements inferred along branches within the evolutionary tree. The precomputed nature of the Magnum resource is critical for meeting its intended uses but also imposes certain constraints.

### Application to example questions

The present study interrogated a subset of replacements involving three nucleotide substitutions, a buried site in the protein structure, and a short branch in the phylogenetic tree. It is a rare and interesting event when a site experiences multiple amino acid replacements on a short branch. We hypothesized that these replacements on relatively short branches may indicate a functionally important site. The rationale behind this hypothesis is that if none of the intermediates are observed, a "non-Markovian" process might drive the introduction of the second and third transversion after the first occurs, because the amino acids that are intermediates in the F-to-E, F-to-Q, or F-to-K replacements create selective disadvantage on the host organisms. As a counterhypothesis, some of these events correspond to sites that are unimportant for functional reasons that would constrain their evolution, where the intermediates are not seen in the reconstructed history simply because the branch is not articulated (noting the contingencies discussed in the introduction).

The F-to-Q replacement at alignment site 7 along the branch leading to the mammalian retinol binding proteins was a case of poor articulation. Orthologs from the recently completed chicken and frog genomes possessed Tyr, an intermediate amino acid in the F-to-Q pathway. Nevertheless, the structural position of this site suggested that the amino acid residue it holds can modulate ligand binding affinity by influencing the orientation of the dual hydrogen bond donor/acceptor residue at a second site, which is in direct contact with the polar end of the bound ligand. These hypotheses are specific enough to be experimentally testable.

In the same protein family, we noticed an unusual protein sequence from the gecko (*Lygodactylus picturalis*). Literature searches revealed that it had been found in the eye lens and prompted us to ask if the sites replaced along this branch might explain how this protein had been recruited to function as a crystallin. Many of the sites were clustered in a region of the protein with their side chains protruding away from the surface – a pattern consistent with the emergence of a new binding site.

These three examples illustrate how the naturally organized Magnum database supports querying across many protein families, as well as opportunistically following leads obtained by browsing, to quickly explore and generate hypotheses related to protein structure and function.

### Integrating protein evolution and structures

Existing protein sequence and structure data sources are Magnum's foundation, yet Magnum is much more than the sum of its source data. The specific sources of raw data chosen for inclusion in Magnum, and three major steps employed to generate the final product, distinguish Magnum from other databases such as PFAM [38], Pandit [39], and PALI [40]. The Magnum source data is first organized under a common schema designed to allow storage, integration, and retrieval of the source data and its derived information. Importantly, this step establishes the orthologous relationships between sequence and structure protein sites. Genealogies are then built for each protein family and the derived molecular evolutionary information stored in the database. This process is not a trivial one. It requires extensive planning, computational processing, and integrating of results from different stages of the evolutionary analysis. Finally, interactive browsing and analysis tools are deployed that make use of a custom application programming interface to the Magnum database.

The chemical behaviors of polypeptides determine, to a large extent, biological phenotypes. Investigating the molecular basis of phenotypes would be simple if the behavior of a protein and the consequence of replacing residues at particular protein sites were predictable from the linear sequence of amino acids. In the absence of these predictive powers, combining phylogenetic sequence analyses with additional sources of complementary data represents a powerful approach to the study of protein molecular function [41,42]. While many would agree that integrative biology is a worthwhile goal, the slow pace of progress in this direction indicates significant challenges exist. Here we succeeded at developing an integration scheme for just two fields of protein study, evolution and structure, which obviously have a synergistic relationship. We applied the method in a high-throughput manner to full-length protein families where at lea one crystal structure could be identified.

We attribute the successful synthesis, at least in part, to the particularly detailed approach employed for reconstructing protein evolution. While many recognize that homologous sequences are more valuable when aligned and organized phylogenetically, few have considered the benefits of capturing branch-specific replacement patterns through the use of ancestral sequences. Reconstructing amino acid replacements along phylogenetic lineages is a realistic and efficient means of modeling the actual events that have occurred during the divergent evolution of the homologous protein sequences. The alternative, when ancestral sequences are unavailable, is to make 'leaf-leaf' comparisons. In leaf-leaf comparisons lineages are visited multiple times as the paths for different leaf-leaf pairs cross many of the same branches [43]. Leaf-leaf comparisons introduce bias in the counting of replacements, thus making the interpretations more difficult. In addition, leaf-leaf pairs are always separated by at least two branches and the number of replacements separating them will always be larger (in many cases much larger) than the number of replacements along individual branches. When searching for a few replacements of functional importance amidst a background of functionally neutral replacements the smaller groups of mutations organized by node-node comparisons are expected to be helpful. In addition, node-node comparisons should capture multiple substitutions at a site better than leaf-leaf treatments.

Homologous proteins with detectable sequence identity possess nearly identical backbone structures [44]. Therefore, a single structure reasonably depicts the fold of all members of a family of homologs. Here the definition of what constitutes a protein family becomes important. Homeomorphic protein families, such as those from the PIR Superfamily resource, contain similar domain contents and arrangements, and the aligned sites share common structural environments. From a folded protein model, sites with buried residues and sites with residues exposed to the solvent are easily identified. Buried residues commonly interact to stabilize the protein fold, whereas exposed residues can bind crucial ligands, catalyze reactions, and mediate the formation of inter-molecular complexes. Three-dimensional structures also divulge groups of sites that might be distant in a linear sequence but proximal in the folded structure, and regions of the molecule that tend to remain flexible. When different proteins are crystallized as a complex, a detailed map of where and how different proteins interact is also captured. All of these structural insights can be investigated from a fresh perspective when the different levels of protein structure (primary, secondary, etc.) are connected with a detailed account of protein evolution.

### Strengths and weaknesses of the approach

As demonstrated here with examples from the cellular retinoid binding proteins, Magnum allows any inquisitive user to ask questions about individual protein families and get quick answers, which may beg additional questions that are either answerable through Magnum or by consulting the scientific literature. There is no need to be an expert or to spend days or weeks preparing the data before the question can be asked. This is the most obvious advantage of a precomputed resource such as Magnum.

Another distinguishing feature of Magnum is its inclusion of detailed molecular evolutionary and structural information for a large number of diverse protein families. This permits the user to browse or query the data across a range of scales, such as specific replacement patterns (e.g. F-to-Q), groups of coincident replacements along a branch, replacements in similar structural elements, or all of these in combination. For those who are willing to follow leads opportunistically, Magnum offers a unique resource that enables discovery through browsing. We have found that this can be an effective method for research project selection.

Ideally, Magnum should consider all families of proteins, regardless of whether they are represented in the crystallographic database, and should be rebuilt continuously. Limited resources required us to consider only those families represented in the PDB by a crystal structure, and to construct only a static database from a single release of iProClass (Version 2.62). The first limitation is not excessively severe; as such families are most susceptible to the combination of structural biology and evolutionary biology that has proven to be so valuable in inferring function. The second is more severe, as is illustrated by our discussion of the cellular retinol binding proteins.

Use of Magnum to analyze cellular retinoid binding proteins, and proteins involved in inflammation (M.B. and S.A, in preparation), showed certain deficiencies related to statically built databases. Most notable of these was the absence of sequences deposited in the public databases since the last build of Magnum was completed.

Another disadvantage is that specific metatheories must be chosen before the database is built. The metatheories and evolutionary parameters that we chose here generated outputs that appear, by various metrics, reasonably accurate, meaning that errors in the alignments, trees, and ancestral sequences will not dominate any conclusions drawn by automated analysis of the database. Ideally, if resources were available, the evolutionary models for individual families would also be continuously upgraded as these metatheories improve. We can even imagine a human inspector evaluating each protein family.

### Expediency of ancestral sequences

The core of an evolutionary model is a multiple sequence alignment and an evolutionary tree. The first describes the historical relation between individual codons in the genes that encode the derived proteins; aligning two amino acids in two derived sequences is equivalent to a statement that the codons encoding these two amino acids are descendents of a single codon in the last common ancestral gene for the two.

Few dispute the existence of the ancestral gene/protein, given the existing derived genes. The likelihood that the model for any particular ancestral sequence corresponds to a sequence that actually existed depends on many factors. Most critical are the number of homologous sequences that contribute to the reconstruction, the extent to which the sequences have diverged, and how the tree connecting them is articulated. Perhaps obviously, more sequences, less divergence, and greater articulation leads generally to a higher likelihood of correspondence than fewer sequences, more divergence, and less articulation. Also obviously, correspondence requires that the multiple sequence alignment and the phylogenetic tree represent the true evolutionary relationships. The treatment of phylogenetic correctness has received little attention in this study for several reasons. The true evolutionary relationships for a protein family, including topology and branch lengths, are unknowable in many cases due to the large number of possible trees. Assessing the reliability of an evolutionary tree through bootstrapping is also not without problems [45]. These issues would be partially surmountable if additional resources were available. For example, the hierarchal Bayesian framework integrates over uncertainty in the tree and branch lengths when inferring ancestral sequences [46].

Given a substantial dataset and satisfactory congruence between the actual history of a protein family and features of the model for that history, other factors become important. These include the choice of a metatheory describing the likelihood of specific amino acid replacements and various tactics for apportioning variation between different sites. Although much has been written comparing these various features of evolutionary metatheories (e.g. [47]), the selection of a metatheory generally has far less impact on the outcome than other factors. In particular, it does not appear to be important to consider higher order models for protein sequence evolution that include replacements at adjacent or distant sites when inferring the amino acids at individual sites in ancestral sequences. To a good approximation, the amino acids at the derived sites are sufficient [48-50].

## Conclusion

In these demonstrations we have relied upon universal protein/codon relationships, and additional criteria from structural and evolutionary biology, to investigate individual amino acid replacements requiring three nucleotide substitutions. We remind that this is but one of many analyses that can be performed using the protein information assembled in the current version of Magnum. Any analysis that considers how the amino acid replacements recorded at individual sites, or collections thereof, along a single branch (or even branches in series) have affected a measurable property of the descendent protein(s) would be addressable with Magnum. Of course, analyses that integrate structural information would make fullest use of the resources delivered.

The integrative framework outlined here can be viewed as the outset of an ambitious project to model biological systems from the bottom up. Integrating biological data from other maturing areas would increase the number and type of questions that can be addressed. For example, additional molecular components of biological systems (e.g. transcription, translation, and protein dynamics), connections between these components (e.g. biochemical pathways, protein interactions, and metabolic networks), and even a dating framework for inferring correlations between the molecular and natural history records could be integrated. When done properly, the integration of disparate, but interrelated, sources of biological information offers a powerful resource for asking biological questions, where the answers can be used to formulate new hypotheses and make predictions that warrant experimental testing.

## Methods

### Protein families and structures

Sequences and annotations for a universal, non-redundant protein set were obtained from the iProClass resource [51]. Version 2.62 of iProClass was obtained in March 2005. PIR Superfamily classifications [25] were also obtained from iProClass. Unlike protein family classifications that split individual proteins into domains, PIR Superfamilies include full-length proteins with common domain architectures and sequence similarities over their entire lengths.

To manage the redundancy in the Protein Data Bank, a non-redundant set of protein chains from structures solved by crystallography was obtained from the Pisces protein culling server [52]. Each protein chain in the PDB was then associated with individual iProClass protein entries. This was accomplished with data from the Seq2Struct resource [53], which associates proteins in the PDB to entries in Swiss-Prot and TrEMBL [54].

In this way we obtained a set of PIR Superfamilies containing at least one member associated with a structure in the PDB. Secondary structure data were obtained using the dictionary of protein secondary structure (DSSP) [55]. Surface accessibility of individual residues is reported as the DSSP value divided by the maximum value obtainable for the residue [56]. When more than one PDB chain is aligned at a site we report the average surface accessibility. Atomic coordinates were collected from PDB legacy files.

### Multiple sequence alignments

To begin investigating families and structures together, global multiple sequence alignments of family members and PDB chains were constructed using ClustalW [57]. Because insertions and deletions occur less frequently in structured (alpha helix or beta sheet) regions relative to unstructured regions [58], secondary structure information was used to set the scoring penalties for placing gap characters in the alignment.

For each family, a second alignment was constructed that corresponds to the region in between the first and last columns having homology to at least one PDB chain residue. Redundant sequences were removed from these alignments. Sequences with fewer characters than 60% of the alignment length were deemed to be fragmentary and were also removed from the alignment. These alignments were used for all further analyses.

### Amino acid replacement model

Pairwise distance estimation, phylogenetic tree inference, and ancestral sequence reconstructions were carried out with the Ancescon package [50]. Ancescon employs a probabilistic model of amino acid replacement in which alignment sites are assumed to evolve independently according to a homogeneous, stationary and time reversible Markov process. The assumption of time reversibility dictates that the matrix of amino acid substitution probabilities (or relative rates), S, be symmetrical. The S matrix used was collected from more than 3,000 globular sequences and is one of the best available descriptions of amino acid replacement for most protein families [59].

For each alignment, an amino acid frequency vector, π, and a rate matrix, **Q**, are determined. Off diagonal elements Q*ij *are calculated as the off diagonal elements of the matrix product

**Q **= S * *diag*(π)     (1)

and diagonal elements *Q*_*ii *_were fixed so that the row sums of **Q **equal zero. In theory, the transition probability matrix of all 20 amino acids

**P**(t) = *e*^**Q**t ^    (2)

describes the probability of amino acid *i *being replaced by amino acid *j *over time *t *as *P*_*ij*_(*t*). In practice, *P*_*ij*_(*t*) values are calculated by decomposing the **Q **matrix into eigensystems.

### Substitution rate factors, pairwise distances, and phylogenies

For each site, a substitution rate factor was calculated using the alignment based method [50]. Invariant sites were assigned a rate of zero. At variable sites, substitution rates were calculated according to the average transition probability for all non-gapped, non-identical amino acids pairs and normalized by the length of the alignment. In this way, the substitution rate across all alignment sites sums to the length of the alignment, just as if the substitution rate per site was held at unity.

Evolutionary distances between leaf sequences were estimated by maximum likelihood while incorporating substitution rate factors for each site. From the matrix of leaf-leaf distances, phylogenetic trees were inferred with the weighted neighbor joining algorithm, Weighbor [28]. Weighbor accounts for the larger errors inherent in longer distance estimates and has been reported to escape the problems of long branches experienced by parsimony and neighbor joining approaches. Weighbor produces trees comparable to those from ML phylogeny reconstruction in much less time.

### Ancestral sequence reconstructions

Probabilistic ancestral sequences for each internal node were inferred using the Ancescon package [50]. The models implemented in Ancescon deal with each site in an alignment independently. The only model variables are the ancestral amino acids. Other parameters in the model are defined for each family. These include an amino acid frequency vector calculated from the alignment, a set of branch lengths from Weighbor, substitution rate factors for each site, and, of course, the observed leaf sequences. Ancestral amino acids at a site are reconstructed by maximizing the conditional probability of the ancestral character given the observed data in the leaf sequences. Reconstruction using pre-set model parameters is generally referred to as an empirical Bayesian analysis [46].

Ancescon implements the method of Koshi and Goldstein [48] for marginal reconstructions. In marginal reconstruction, each internal tree node site is treated separately. For each site at an internal node, the probability of ancestral amino acid X (A_X_) conditional on the set of observed leaf sequences {A_L_}, the mutation matrix (M), and the phylogenetic tree (T) is calculated using Bayes' Theorem:

P(AX|AL,M,T)=P({AL}|AX,M,T)*P(AX)P({AL}|M,T)     (3)
 MathType@MTEF@5@5@+=feaafiart1ev1aaatCvAUfKttLearuWrP9MDH5MBPbIqV92AaeXatLxBI9gBaebbnrfifHhDYfgasaacH8akY=wiFfYdH8Gipec8Eeeu0xXdbba9frFj0=OqFfea0dXdd9vqai=hGuQ8kuc9pgc9s8qqaq=dirpe0xb9q8qiLsFr0=vr0=vr0dc8meaabaqaciaacaGaaeqabaqabeGadaaakeaacqqGqbaucqGGOaakcqqGbbqqdaWgaaWcbaGaeeiwaGfabeaakiabcYha8jabbgeabnaaBaaaleaacqqGmbataeqaaOGaeiilaWIaeeyta0KaeiilaWIaeeivaqLaeiykaKIaeyypa0ZaaSaaaeaacqqGqbaucqGGOaakcqGG7bWEcqqGbbqqdaWgaaWcbaGaeeitaWeabeaakiabc2ha9jabcYha8jabbgeabnaaBaaaleaacqqGybawaeqaaOGaeiilaWIaeeyta0KaeiilaWIaeeivaqLaeiykaKIaeiOkaOIaeeiuaaLaeiikaGIaeeyqae0aaSbaaSqaaiabbIfaybqabaGccqGGPaqkaeaacqqGqbaucqGGOaakcqGG7bWEcqqGbbqqdaWgaaWcbaGaeeitaWeabeaakiabc2ha9jabcYha8jabb2eanjabcYcaSiabbsfaujabcMcaPaaacaWLjaGaaCzcamaabmaabaGaeG4mamdacaGLOaGaayzkaaaaaa@620F@

where the P({A_L_} | A_X_, M, T) is the conditional probability of the leaf sequences for a given ancestral amino acid, and P(A_X_) the probability of the given ancestral amino acid. Because the amino acids at all other internal nodes are unknown, P({A_L_} | A_X_, M, T) is obtained by summing the probabilities of the replacements needed to generate the leaf sequences through all possible replacement paths between the node and the leaf sequences. For example, for a single site at an internal node in an unrooted tree with four leaf sequences (1–4) and two internal nodes (5, 6), the P({A_L_} | A_X_, M, T) can be expanded as:

P(A1,A2,A3,A4|A5,M,T)=∑A6PA5A6(αi⋅dA5A6)PA5A1(αi⋅dA5A1)PA5A2(αi⋅dA5A2)PA6A3(αi⋅dA6A3)PA6A4(αi⋅dA6A4)     (4)
 MathType@MTEF@5@5@+=feaafiart1ev1aaatCvAUfKttLearuWrP9MDH5MBPbIqV92AaeXatLxBI9gBaebbnrfifHhDYfgasaacH8akY=wiFfYdH8Gipec8Eeeu0xXdbba9frFj0=OqFfea0dXdd9vqai=hGuQ8kuc9pgc9s8qqaq=dirpe0xb9q8qiLsFr0=vr0=vr0dc8meaabaqaciaacaGaaeqabaqabeGadaaakeaacqWGqbaucqGGOaakcqWGbbqqdaWgaaWcbaGaeGymaedabeaakiabcYcaSiabdgeabnaaBaaaleaacqaIYaGmaeqaaOGaeiilaWIaemyqae0aaSbaaSqaaiabiodaZaqabaGccqGGSaalcqWGbbqqdaWgaaWcbaGaeGinaqdabeaakiabcYha8jabdgeabnaaBaaaleaacqaI1aqnaeqaaOGaeiilaWIaemyta0KaeiilaWIaemivaqLaeiykaKIaeyypa0ZaaabuaeaacqWGqbaudaWgaaWcbaGaemyqae0aaSbaaWqaaiabiwda1aqabaWccqWGbbqqdaWgaaadbaGaeGOnaydabeaaaSqabaaabaGaemyqae0aaSbaaWqaaiabiAda2aqabaaaleqaniabggHiLdGccqGGOaakcqaHXoqydaWgaaWcbaGaemyAaKgabeaakiabgwSixlabdsgaKnaaBaaaleaacqWGbbqqdaWgaaadbaGaeGynaudabeaaliabdgeabnaaBaaameaacqaI2aGnaeqaaaWcbeaakiabcMcaPiabdcfaqnaaBaaaleaacqWGbbqqdaWgaaadbaGaeGynaudabeaaliabdgeabnaaBaaameaacqaIXaqmaeqaaaWcbeaakiabcIcaOiabeg7aHnaaBaaaleaacqWGPbqAaeqaaOGaeyyXICTaemizaq2aaSbaaSqaaiabdgeabnaaBaaameaacqaI1aqnaeqaaSGaemyqae0aaSbaaWqaaiabigdaXaqabaaaleqaaOGaeiykaKIaemiuaa1aaSbaaSqaaiabdgeabnaaBaaameaacqaI1aqnaeqaaSGaemyqae0aaSbaaWqaaiabikdaYaqabaaaleqaaOGaeiikaGIaeqySde2aaSbaaSqaaiabdMgaPbqabaGccqGHflY1cqWGKbazdaWgaaWcbaGaemyqae0aaSbaaWqaaiabiwda1aqabaWccqWGbbqqdaWgaaadbaGaeGOmaidabeaaaSqabaGccqGGPaqkcqWGqbaudaWgaaWcbaGaemyqae0aaSbaaWqaaiabiAda2aqabaWccqWGbbqqdaWgaaadbaGaeG4mamdabeaaaSqabaGccqGGOaakcqaHXoqydaWgaaWcbaGaemyAaKgabeaakiabgwSixlabdsgaKnaaBaaaleaacqWGbbqqdaWgaaadbaGaeGOnaydabeaaliabdgeabnaaBaaameaacqaIZaWmaeqaaaWcbeaakiabcMcaPiabdcfaqnaaBaaaleaacqWGbbqqdaWgaaadbaGaeGOnaydabeaaliabdgeabnaaBaaameaacqaI0aanaeqaaaWcbeaakiabcIcaOiabeg7aHnaaBaaaleaacqWGPbqAaeqaaOGaeyyXICTaemizaq2aaSbaaSqaaiabdgeabnaaBaaameaacqaI2aGnaeqaaSGaemyqae0aaSbaaWqaaiabisda0aqabaaaleqaaOGaeiykaKIaaCzcaiaaxMaadaqadaqaaiabisda0aGaayjkaiaawMcaaaaa@AABC@

where the summation is over each of the twenty amino acids at node A_6_. For each site at internal node A_5_, the P(A_X _| A_L_, M, T) is calculated for each of the twenty amino acids. Thus, at each internal node site marginal reconstructions generate a vector composed of the twenty amino acids and their posterior probabilities. In this context, the best ancestral amino acid at a site will be that which has the highest posterior probability.

Ancescon implements the method of Pupko et al. [60] for joint reconstructions. In a joint reconstruction sites are treated independently but all internal nodes are treated as a set. The objective is to identify the most likely set of amino acids for all internal nodes at a site that yields the maximum joint likelihood of the tree. As a result, only a single amino acid is reported for each site and conditional posterior probabilities at a site are not available. For details of the joint method the reader is referred to [60].

Gaps were placed in ancestral sequences according to the default settings of the Ancescon program. The same rule-based strategy was applied in both marginal and joint reconstructions. Thus, the gapping history is the same regardless of the reconstruction method. The placement of gaps in ancestral sequence proceeds by traversing a tree from the leaves inward. If an internal node had one or zero branches leading to the remaining descendents a gap character was placed at that site at that node.

### Reconstructed amino acid replacements

Replacements were tallied by comparing residues at aligned sites of parent (ancestor) and child (descendent) sequences that define a branch. Note that the parent-child relations are created for organizational purposes and do not imply the use of rooted phylogenies. Three methods of counting were employed. The simplest method [61] counts a replacement with weight probability of one if the best parent and child amino acids are different. This is referred to as the 'best count' method. The best residue was judged by the posterior probability values for all twenty residues. The 'best fractional' method finds replacements by the same rule as the best count, but counts are given a weight probability equal to the product of the parent and child posterior probabilities. The 'all fractional' method compares each parent with each child residue and reports the sum of combined probabilities where the amino acids differ. In the rare event that two residues had the same posterior probability the residue suggested by the Ancescon software was treated as the primary residue. Note that these ties only occur when the best possible residue has a posterior probability less than or equal to 0.5.

### Count and Dayhoff matrices

Log-odds (Dayhoff) matrices were created with the *CreateDayMatrices *option in the DARWIN package [62]. As input, count matrices were constructed using the three counting methods described above. For the 'best count' method, identical amino acid pairs (on-diagonal terms) were scored with a value of 1. A value of 0.5 was added to both off-diagonal elements for non-identical pairs [61]. For example, upon observing a TS (threonine-serine) pair, 0.5 was added to both TS and ST matrix elements. This results in a symmetrical matrix to account for the fact that the direction of change is unspecified along a branch in an unrooted tree. In the matrices derived with 'best fractional' and 'all fractional' tools, pairs were scored as the product of the posterior probabilities. As before, non-identical pair scores were halved and added to both matrix elements.

Count matrices were constructed from pairs of aligned sequences representing either two consecutive internal nodes (node-node), or two leaf sequences (leaf-leaf). Using branch length values (*l*), sequence pairs were placed into one of three bins, short (0.005<*l *<0.015), medium (0.120<*l *<0.220), and long (0.520<*l *<1.00). In each range, 10,000 node-node or leaf-leaf sequence pairs were randomly selected. For node-node pairs the branch lengths were the values from the Weighbor phylogenies. For leaf-leaf pairs, branch lengths represent the sum of individual branch lengths along the shortest path from one leaf to another. The chosen ranges were a compromise between maintaining a narrow enough range to keep the members of each bin similar, while maintaining a wide enough range to obtain enough branches to permit statistical analyses. Frequency counts and Dayhoff matrices are provided as supplementary material [see Additional file 1].

## Availability

The Magnum home page on the internet  contains a family search interface, and generates dynamic pages for each family. This environment is well-suited for interactive inspection of the protein members, multiple sequence alignments, phylogenetic trees, crystal structures, all of the F-to-E/K/Q replacements, as well as other features of the protein families. The PERL application programming interface is also obtainable from the Magnum home page. The MySQL database tables are currently being distributed on digital video discs upon request from the authors.

## Authors' contributions

MB performed the study and drafted the manuscript. SB participated in designing the study and preparing the manuscript.

## Supplementary Material

Additional File 1 Frequency counts and Dayhoff matrices. Frequency counts of amino acid pairs from bins of short, medium and long branches, and Dayhoff log-odds matrices derived from these counts. Click here for file

Additional File 2 F-to-E/Q/K replacements. List of F-to-E/Q/K replacements on short branches and at buried sites. Click here for file

Additional File 3 Multiple sequence alignment of cellular retinoid binding proteins. Aligned sequences ordered according to the tree shown in Figure 6. The sequence of the reference structure PDB:1opbA has been underlined. Click here for file

Additional File 4 Amino acid replacement report. Amino acid replacements for the seven labeled branches in Figure 6. Click here for file
