# Supplementary material for: Integrating protein structures and precomputed genealogies in the Magnum database: Examples with cellular retinoid binding proteins
Source: BMC Bioinformatics. 2006 Feb 23;7:89. doi: 10.1186/1471-2105-7-89 (PMC1475641; doi:10.1186/1471-2105-7-89)
Supplement: Additional File 1 — Frequency counts and Dayhoff matrices. Frequency counts of amino acid pairs from bins of short, medium and long branches, and Dayhoff log-odds matrices derived from these counts. [file 1471-2105-7-89-S1.pdf]

Dataset S1. Frequency counts and Dayhoff matrices for short, medium and long branches.

Frequency counts for all 210 unique amino acid pairs

	Leaf-Leaf			Node-Node (All Fractional)			Node-Node (Best Fractional)			Node-Node (Best Count)		
**ij**	**Short**	**Medium**	**Long**	**Short**	**Medium**	**Long**	**Short**	**Medium**	**Long**	**Short**	**Medium**	**Long**
CC	52607	34836	24992	41905.7	26765.1	18329.5	41795.7	26175.4	17413.6	42184	27580	18590
SC	87.5	519	3015.5	164	777.5	1416.1	87.5	413.1	891.7	104.5	603	1319
SS	206697	107262	84844	165091	113228	72445.5	163600	105470	62876.7	171703	132798	80683
TC	14	142.5	2188	54.1	376.2	890	20.9	183.1	577.8	26	262.5	844
TS	669.5	4891	15504.5	2047.9	9489.1	12876.7	419.4	3098.4	6977.4	607	5471	10823
TT	200431	75164	93865	155034	114078	71281.8	153909	108368	64420.8	159793	129646	78695
PC	10	35.5	305	9.8	59.5	242.9	6.8	30.6	170.2	8.5	45	243.5
PS	479.5	2345.5	4649	552.5	2857.7	4896.3	182.7	1010.2	2832.3	239	1843.5	4489
PT	98.5	1083	3580	197.8	1344.4	2787.7	63.5	467.6	1605.1	81	860.5	2584.5
PP	164186	90828	101444	133844	123587	90840.9	133443	121081	87705.3	135747	131822	95707
AC	16	267	4909	126.2	1093.7	2388.1	37.7	596.6	1715.5	55	863.5	2366.5
AS	593	5112.5	23202.5	2491.3	11729.4	15991.9	520.3	3831.2	8756.4	764.5	6688.5	13418
AT	857	4133	11184	1377.2	6271.6	9276.4	420.7	1914.6	4728.4	560	3534.5	7748.5
AP	170	2451	7393	674.2	3829.8	6469.8	185.9	1556.4	4057.2	250	2588	6124
AA	246033	118620	156874	229575	204727	134964	227909	195807	124226	237037	231117	149212
GC	62	110	1321	21.1	176.2	563.9	12.6	105.8	417.1	15	145.5	554
GS	324.5	4716.5	8601	642.8	3550	6708.1	226.4	1448.6	3958.8	303	2449.5	6025
GT	13	343	3452.5	73.1	768.7	2307.1	23.6	268.3	1256	34	464.5	2035.5
GP	29	295.5	2508	64.4	670.4	2152.4	24.7	322.8	1510.4	30.5	511	2193.5
GA	344.5	3441.5	13274.5	827.9	5326.1	10600.6	285.5	2595	7237.8	380.5	3867	10084
GG	241004	113154	203713	215438	199845	154633	214947	196914	150484	217275	206898	159611
NC	5	57	1036.5	14.4	140.5	345.1	6.6	74.9	195.6	9	106.5	312.5
NS	633	4561.5	8030	1397.1	5751.5	7750.2	356.2	1592.7	3546.8	484	3187	6235.5
NT	200.5	1037.5	5793	539.2	2891.3	4563.6	139.6	886.7	2154.2	195.5	1706.5	3749
NP	10	125	2005.5	50.5	557.5	1554.7	11.2	147.3	813.7	14.5	293	1353
NA	15.5	580.5	5147	250.6	1944.1	3679.1	30	444	1552.6	54.5	951	2900
NG	25	1033	7585.5	374.7	2598.4	4824.6	90.7	1130.6	2975.9	136	1847.5	4434.5
NN	155105	53833	70025	118624	82842.8	53100.8	117593	77580.6	46906.2	122957	95596	58186
DC	7.5	36	293	7.5	46.4	197.9	5.8	24.6	113.6	6.5	37	178.5
DS	51.5	1125	5958.5	415.4	3096.4	6047.6	71.7	967	3035.9	120.5	1957	5317
DT	13	263.5	3918.5	116.9	1325.5	3238.4	21.8	405	1583.1	33.5	782	2796.5
DP	11	189.5	2269.5	111.1	1152.3	2812.6	24.1	471.6	1756.4	37	827	2767
DA	86.5	1616.5	5896	351.2	2704.1	5205.7	77.1	820.4	2411.6	115.5	1586	4387.5
DG	273	1524.5	5953.5	365.3	2338.9	5058.5	144	1045.8	3204.8	185.5	1705	4778
DN	399	2158	11830	1541.3	7627.3	10425.9	329.2	2336.6	5279.7	464.5	4257.5	8436
DD	198101	86557	102407	161649	134069	93112.5	160400	127252	84781.9	166097	149298	100835
EC	1	43.5	359	4	52.5	249.6	2.7	21.2	127.7	3.5	39	229.5
ES	11	544	7543	463.3	3358.6	6599.2	38.5	816.2	3265.8	77	1880	6154
ET	16.5	377	6132.5	389.9	2768.6	5270.8	44.6	849.3	2770.9	80.5	1786	5102.5
EP	10.5	563	3727.5	261	2094.7	4234.6	36.2	790.7	2640.7	61.5	1507.5	4340
EA	139.5	1601.5	11641.5	1297.9	7335.6	10974.1	189.5	2533.1	6083.5	326	4986	10602
EG	252	1243	5296.5	290.9	1851.3	4440.4	118.1	720	2645.8	153.5	1281.5	4364.5
EN	24	355	7587	497.9	3337.4	5739.4	40.4	627.5	2455.3	87	1497	4763.5

ED	1059	9118	23994	3575.8	16313.5	21060.1	662.1	4871.6	11375	974	8709	18156.5
EE	216480	127534	130829	197686	165765	104724	195887	155974	94312.8	207584	201060	122816
QC	2.5	32	330.5	4.7	67.9	203.8	1.3	30.4	95	3	52	173
QS	17	889.5	4424	339.2	2206.7	3533.4	34.7	531.9	1371	62.5	1203.5	2729.5
QT	6	397.5	3841	261.6	1781.4	2837.6	28.7	490.9	1170.9	55	1025	2267
QP	49.5	1283.5	1670.5	178.9	1127.4	2004.9	53.5	365.7	1013	74	713.5	1694.5
QA	27.5	760	6381	469.6	3036.9	4689	54.4	852.4	2043.1	103.5	1809	3848.5
QG	20.5	257	2470	84.5	771	1913.6	22.4	281.4	992.9	31.5	499.5	1627.5
QN	16.5	318.5	4194	367.4	2218.7	3271.1	37.9	555.1	1320.7	72.5	1178.5	2519.5
QD	12	343.5	4778.5	197.2	1936.6	3736.8	21	407.2	1423	41	825	2604
QE	223.5	3450	12728	2159.6	10443.9	12243.4	243.5	2793.4	5944.5	404	5631	10284
QQ	132319	69868	48688	103993	69832.1	37495.8	102796	63655.8	31197.6	109052	83055	41200
HC	0	62.5	256.5	9.8	80.7	199.4	3.2	38.3	118.4	3.5	60	188
HS	12.5	291.5	1981.5	122.4	928.9	1782	16.2	250.8	808.2	27.5	539.5	1516.5
HT	21	168.5	1574.5	74.7	579.8	1267.8	18.6	170.8	622.4	27	354.5	1135
HP	26	680	733	62.1	431	991.5	23	157.2	593.4	29	288.5	935
HA	16.5	127.5	2076.5	77.5	734.1	1615.1	13	239.5	794.4	21	474.5	1456
HG	1	246.5	1540	31.4	366.3	1057.9	9.1	155.7	658.1	15	251.5	1004
HN	155.5	915.5	4428	463.2	2269.4	3151	106	789	1635.4	151.5	1395.5	2627
HD	120	366.5	1682	120.4	902.2	1899.3	41	313.9	997.6	54.5	566	1684.5
HE	8	162	2947	107.1	1005.3	2170	16.9	271.4	1067.6	27	601	1987.5
HQ	194.5	2536.5	2275.5	479.8	2271.1	2902.2	127.4	749	1420.8	169	1346.5	2386.5
HH	80066	50468	34095	65041.6	50691	33914.1	64664.4	48578.1	31433.9	66659	55738	35941
RC	97	160.5	587.5	46	204.4	466.2	31	109.2	287.5	35	161.5	462.5
RS	148	932.5	3413.5	352.5	2432.9	4685.4	76.7	674.8	2216.5	111.5	1437	4079
RT	77	471.5	3194	180.4	1554.9	3349.3	34.1	444.5	1694.5	53.5	922.5	3064
RP	54	1144.5	1523	121.6	1007.1	2456.6	39.8	352.3	1481	52.5	667.5	2373.5
RA	97	425	3838	294.4	2300.5	5107.7	70.2	695.2	2662	96.5	1452	4750.5
RG	194	868.5	2493.5	179.6	1063.7	2912.6	91.7	436.4	1792.1	110.5	774	2804.5
RN	13.5	401	3737	170.2	1524.4	3244.2	24.7	387.7	1485.9	39.5	786	2672
RD	15.5	141	2281.5	50.7	706.3	2438.2	11.3	154.1	1081.1	15.5	330	1984.5
RE	25	327.5	5544.5	212.7	2307.5	6063.6	24.3	562.6	3150.8	41.5	1299.5	5818.5
RQ	192.5	2781	5169	838.7	4714.9	6621.1	143	1254.8	3001.7	205	2501.5	5307
RH	196.5	2005	2278	327.4	1881.2	2995.1	104.9	651.3	1618	137.5	1189.5	2688.5
RR	172819	94200	100073	142489	122700	86004.7	141582	117034	78789.7	146477	137964	94593
KC	0	18	598	3.9	71.9	302.2	0.6	27	159.9	1.5	46	286
KS	22	912	8920	496.4	3308.7	5986.3	57.3	789.3	2761.5	101.5	1839.5	5331
KT	270.5	938	8963	572.8	3425.1	5504.7	100.4	1101.3	2807.4	166.5	2242	5093.5
KP	6.5	424.5	5055	175.5	1497.8	3264.4	33.2	563.2	2001.1	53	1051.5	3253.5
KA	12.5	440.5	13461	616.9	4087.5	7048.8	67.9	1236.5	3554.1	136	2643	6503.5
KG	17.5	324.5	7252.5	139.2	1277	3431.2	38.8	493.7	2064.5	60	888.5	3304.5
KN	381.5	2180.5	9094	972.3	4781.3	6923.3	200.1	1346.4	3346.9	286	2791	6100
KD	15	304	7720.5	215.3	2180	5097.2	29.5	490.9	2311.7	51.5	1059.5	4343
KE	399.5	1781	19094.5	1688.4	9325.4	14079.4	222.3	2620.9	7706.1	373	5651	13733
KQ	110.5	2808.5	12646	1757.8	7935.8	8933.6	202.6	1874.9	3863	328	4000	7026.5
KH	3	276.5	4270.5	157.5	1286.8	2384.6	21.1	343.2	1143.3	34	719	2094
KR	669.5	7743.5	21203	2688.2	13460.2	18068.6	451.4	4085.1	9798.9	663	7377	15364
KK	209417	126820	111614	174638	128458	80529.2	173081	119955	70984	182792	156613	94377
MC	2	25.5	561	10.5	108.3	269	3.2	47.4	147.6	4.5	72	221
MS	6	241.5	1264	73.8	530.7	1127.5	10.3	140.6	497.1	18	306.5	897
MT	101	1220	1919.5	243.6	1132.7	1810.6	77.4	393.8	928.2	103.5	719.5	1557
MP	4.5	81.5	665	20.7	185.8	554.5	6.4	66.3	318.4	9	117.5	500
MA	8	224	2577	153.4	1215.2	2377	23.4	441.5	1277.8	43	813	2079.5
MG	8	113.5	604	18.8	162.1	550.8	9.5	64.9	312.9	12.5	119.5	485.5

MN	18	46.5	808.5	30.5	221.6	570.7	10.6	64.7	249.8	13	132	453.5
MD	0	35	441	8.6	123.5	468.6	0.9	31.6	203.2	1.5	70.5	368.5
ME	7.5	81.5	1159	40.8	412.9	1041.4	5.2	128	500	10	272	919.5
MQ	5.5	314.5	1351.5	132	790.8	1284.4	23.4	274.2	635	37.5	516.5	1057
MH	2	41	599	19.5	173.4	435.3	3.4	44.1	211.7	5.5	93.5	361.5
MR	17.5	400	1094.5	77.4	549.2	1161.7	17.6	157.8	570.4	26.5	322.5	990
MK	65.5	300.5	1749	120	778.6	1494.4	27.9	260.5	751.1	45.5	505.5	1298.5
MM	88616	31777	35927	66635.1	47661.2	26137.2	66144.7	45021.3	23116.5	68492	52276	27183
IC	12	47	1182.5	17.7	173.4	594.1	8.4	72.9	311.5	10	112	461
IS	36.5	226.5	2029.5	65.1	607.5	1866.7	23.6	144	745.2	29.5	305	1407.5
IT	270	2193.5	5618.5	415.8	2266.2	4402.1	154.1	854.8	2329	204.5	1510	3824.5
IP	12	120	1421	23.4	295.9	1155.1	8.5	106.8	695.1	11.5	181.5	1041.5
IA	24.5	707	5626.5	102.4	1359.5	4220.4	19.8	380.6	2120.1	30.5	682	3423
IG	12.5	111	960.5	9.3	124.6	681.7	5.5	41.4	347.1	6.5	71	545.5
IN	47.5	162.5	1607	60.6	444.7	1233.2	27.1	153.7	542.3	38.5	292	981.5
ID	9.5	53	961	13.3	151.9	775.9	6.3	32.9	313.3	8.5	67.5	577
IE	18	116	2702	39.9	493.2	1805.9	12.2	145.6	875	18.5	292	1634.5
IQ	2.5	95	1734	29.4	391.1	1208.6	3.5	88.5	467.8	6	187	884
IH	8.5	45.5	1003	17.1	200.8	643	4.5	59.3	297.6	7	114.5	526.5
IR	35	89	1762.5	42.7	497.2	1683.7	12.4	138.8	848.8	18.5	288	1466
IK	10.5	314.5	3449	80.2	766.3	2223	24.5	270.9	1123.9	38	519	2096
IM	243.5	1615	7151.5	560.4	3005.4	4674.4	144.3	996.9	2439.6	196.5	1657	3682
II	173091	89259	123270	167577	139209	95225.4	166164	131014	84645	172223	153911	101303
LC	17	126	1642.5	33.9	360.8	1080.4	14.1	196.6	763	18.5	302.5	1107.5
LS	135	695	2745	160.2	1125.2	3002	64.7	354.8	1448.3	84	746	2677
LT	21.5	472	4726.5	158.8	1486.4	3945.6	42.2	528.3	2251.6	64.5	1056	3805.5
LP	204.5	1560.5	2573	162.1	942	2625.5	89.5	470.1	1810.3	106	793.5	2723.5
LA	28	565.5	6297.5	238.8	2505.2	7174	59.3	993.6	4538.2	97.5	1880.5	7218.5
LG	12	118.5	1736	22.7	305.5	1396	10.6	146	907.5	14.5	259	1427
LN	15.5	102.5	1958.5	38.4	465.9	1576.4	11.5	145.6	778.7	17	305.5	1447
LD	6.5	125.5	1673	18.2	318.4	1398.5	4.3	106.1	705.8	7	208	1286.5
LE	14.5	188.5	3856.5	68	895.2	3147.3	18	328.1	1802.1	26.5	711	3325
LQ	297	871	3662.5	254.8	1806.8	3518.1	76.3	748.8	1857.7	108.5	1408.5	3295.5
LH	50	701.5	1828	78.7	675.2	1622.6	24.7	257.1	928.4	32.5	484.5	1566
LR	70	467.5	3102	175.3	1588.6	4045.3	52.1	665	2489	77.5	1269	4122.5
LK	14	187	5188.5	126.4	1283.1	3706.8	30.7	476.4	2098.5	49.5	966	3752.5
LM	119	3504.5	12150	1174.4	6648.2	9418.2	234.4	2509.7	5606	333	3895	7992.5
LI	423.5	4449.5	30056	1787.3	12492.7	22687.9	374.6	5036	14530	551.5	7898	20206
LL	322389	187991	192135	260620	234915	173455	259598	228082	163997	265288	256330	188204
VC	26	159	3072	101.7	758.8	1753.2	38.9	421.5	1253.5	52	636	1785
VS	13	457.5	3217	109.1	1128.5	2952.5	20.1	274.8	1339.5	30	583	2396.5
VT	41.5	1948.5	9368	628.1	4090.4	6975.3	118.9	1668.6	4194.8	187	2851	6596.5
VP	9.5	433	1887.5	86.8	829.8	2198	28.5	379.3	1439.4	42	632	2162.5
VA	751	5947	12995	1306.9	7510.5	13130.2	415.1	3232.9	8501.1	558.5	5314.5	12476.5
VG	232	468	1888.5	67.6	434.7	1578.4	44.3	189.7	945.1	50.5	328	1491.5
VN	5.5	233	1719	41.7	487.1	1313.3	7.4	141.3	547.1	12.5	289.5	1056
VD	53	152.5	1288.5	45.1	399.6	1337.2	22.9	128.8	612.9	28	252	1155.5
VE	78	519	4189.5	205.8	1561.8	3490.7	60.5	648.9	1972.2	93	1237	3548
VQ	6.5	125	2282.5	83.4	784.3	1750.4	15.2	226.9	753.2	31.5	458.5	1430
VH	1.5	51.5	958.5	22.9	262	802.8	4.1	75.3	409.7	6	156	740
VR	19.5	131.5	2444	77.2	818.6	2246	18.1	280.2	1192.2	29	565.5	2105.5
VK	20.5	200	5488	134.8	1146.7	2809.3	30.7	394	1458.8	54	806.5	2713.5
VM	161.5	2395.5	4406.5	385.9	2129.5	3504.4	96.1	617.5	1723.9	132.5	1113	2772
VI	1073.5	10399.5	41174.5	4277.7	21090	29607.4	894.9	7138.8	17475.6	1278	11413	24840

VL	380	4207.5	18297.5	1163.9	8433.2	16801.2	274.4	3166.4	10369.6	399	5310.5	15329.5
VV	238229	117757	124823	198691	167654	111421	197161	158886	101010	204772	189098	122390
FC	13	145.5	722.5	21.2	160.4	470.6	11	90.6	328.4	13.5	134.5	459
FS	171.5	416	1449	101.8	559.5	1492.4	57.5	215.6	759.5	69	410	1332
FT	6	71	1095	24.4	299.7	1089.1	5.9	92.5	579.4	9.5	202	973.5
FP	7.5	122	833.5	15.7	186.9	710.3	6.5	82.1	468	9.5	147	709.5
FA	6	157	2364	44.4	557.1	1907.3	12.9	226.3	1137.6	20	407.5	1817.5
FG	7	58.5	673.5	9.5	123.3	604.5	4.8	61.6	405.4	6	94.5	599.5
FN	9.5	62.5	760	19.4	182.1	690.1	10.8	61.2	321.8	12	114.5	580
FD	0	39.5	550	6.3	99.3	523	1.9	32.7	260.8	2.5	63	455.5
FE	5.5	83	1276.5	11.2	182.4	852.2	3.8	66.3	466	6	144	855.5
FQ	2.5	58	813.5	10.8	175.8	619.9	2.1	52.7	261.4	3.5	104	473.5
FH	5.5	148.5	1589	58.8	498.4	1147.2	17.3	218	657.3	26	361	1016
FR	9.5	39	896	16.7	213.6	846.8	6.1	66.4	458.8	8.5	134.5	779.5
FK	2	27	1227	11.9	187.9	851.8	3.2	60.3	443.4	5	124.5	814
FM	5	177	2494	93	819.8	1743.2	19.5	347.7	1025.1	29.5	545	1490
FI	39	340.5	4502.5	173.1	1607.3	4102.8	51.1	689	2557.7	74.5	1117	3680
FL	448.5	2706	10616	642.4	4795.2	10131.6	205.4	2295.8	7132.4	266.5	3421.5	9684
FV	29	399	3591	125.5	1231.8	3383.5	35.8	494.1	2031.3	48	869	3100.5
FF	144998	75879	79896	111760	90495.9	65089.3	111374	87831.1	61252.5	113111	95102	67459
YC	77.5	130	529	34.6	165.7	418.9	23	88.7	299.9	26	133.5	430.5
YS	32	639.5	1416.5	98.3	727.3	1663.8	32.3	237	842.8	46	496	1530
YT	13.5	88.5	1386.5	44.6	400.4	1151.1	16.7	145.8	626.9	23	285	1080
YP	9	24.5	734.5	14.9	210.4	692.7	3.7	96.9	460.6	5	166.5	738
YA	5	200.5	2304	46.8	586.3	1739.9	9.5	237.8	1046.7	17.5	457.5	1732
YG	9	45	705.5	11	150.8	681.8	5.5	63.4	441.6	7	109.5	696.5
YN	54.5	211	1729.5	81.9	656.4	1490.7	25	247.2	786.1	37.5	456	1325.5
YD	85.5	177	836.5	36.4	299.1	947.2	18.3	114.7	516.1	22	215.5	902.5
YE	12.5	69.5	1352	26.9	350.7	1241.3	7.2	130.5	723.7	12	281	1299.5
YQ	0.5	179.5	1126	27.7	314	902.5	5.9	97.3	430	9	200.5	798.5
YH	162.5	1050.5	3927	390.3	1993.7	3093	120.4	839.1	1969.6	160	1304	2813.5
YR	9	90.5	1393.5	50.2	519.2	1483.6	13.5	213.4	912	20.5	414.5	1517.5
YK	3	18	2521	24.6	340.8	1182.4	6.3	119.5	640.8	11.5	238.5	1140.5
YM	3	65.5	877	27	285.5	735.5	6	97	394.9	9	174	633.5
YI	14.5	107	1847	48.1	562.6	1760.9	12.6	210.6	1032.1	17.5	377	1620.5
YL	15.5	213	4006	104.8	1158.2	3288.1	26.9	502.3	2182.5	40	862	3340
YV	8.5	72	2063.5	54.2	616.5	1851.2	12.8	247.5	1106.5	21	470	1795.5
YF	227	2736	13451.5	874	5275	8735.9	242.7	2416	5869	327.5	3385.5	7642.5
YY	119513	61014	67715	95983.2	78958.9	56115.9	95639.3	76813.5	53205.3	97273	84421	59132
WC	17.5	46	77	10.1	38.5	104.9	8.3	20.9	69.7	9	33.5	100.5
WS	4	56.5	318	18.6	164.3	411.7	7.1	65	213.6	9.5	122	371.5
WT	0	17	198.5	5	65.1	249.1	1.5	20.8	136	2	41.5	220
WP	1	3	115	2.9	46.4	186.2	0.9	20.5	125.8	1.5	32.5	194.5
WA	0.5	1.5	316	5.8	117.4	432.7	1.1	46.9	253.6	1.5	87.5	417
WG	15.5	49	252.5	15.1	87.6	303.5	11	43.9	209.7	13	73.5	309
WN	0.5	13	382.5	2.2	45.9	168.8	0	13.3	77.4	0	25.5	132.5
WD	2.5	2.5	190	3.9	52.7	198.9	1.5	19.7	112.3	1.5	35.5	187.5
WE	4.5	8	249	5	85	304.4	1.9	37.6	173.9	2.5	77	309
WQ	2	49	268	7.2	82.5	240.7	2.1	31.6	117.4	3.5	56.5	205.5
WH	0.5	22.5	274	10	100.4	267.5	3	46.8	162.9	4	71.5	245.5
WR	54	664	298.5	39.8	270.1	673.5	21.4	117.5	426.7	24	209	685.5
WK	2.5	11.5	378	6.1	76.4	295.3	2.5	21.4	146.2	3	43.5	257
WM	0.5	14.5	251.5	8.5	96.9	250.6	2.1	40.6	135	3	68.5	203
WI	4	10	255.5	6.8	111.2	412.1	2.2	42.2	227.1	3	72	344.5

WL	10	132	971	43.7	463.5	1297.5	16.8	239.1	936.4	22	369	1334.5
WV	3	43.5	297.5	11.9	151.5	507.8	3.4	59.1	296.7	5.5	106.5	491
WF	5.5	117.5	1621	63	600.5	1429.4	23.7	336.8	1048.5	30.5	456.5	1357
WY	3.5	55	1707.5	71.3	730.6	1600.6	19	380.2	1168.9	26.5	541	1559.5
WW	48178	19704	26032	35256.1	28847.3	20988.1	35208	28416.1	20278.4	35417	29643	21344

Leaf-Leaf Short Branches – Dayhoff Matrix

C	2.8																			
S	-1.8	4.6																		
T	1.3	1.0	3.6																	
P	0.4	1.7	3.3	4.1																
A	-0.9	-2.1	-1.5	-1.9	9.4															
G	-1.1	-4.5	-2.3	-3.4	-2.0	4.7														
N	2.0	-1.9	0.2	0.4	-0.9	-1.7	3.0													
D	0.7	0.1	-1.7	-1.8	-1.0	-3.0	0.7	6.6												
E	-0.3	-3.0	-2.3	-2.1	-1.0	1.6	0.8	-0.5	3.6											
Q	0.8	-3.5	-0.2	-1.7	0.6	1.2	-0.7	-1.4	-0.7	4.8										
H	-1.1	-3.9	-2.1	-3.2	-2.7	4.0	-1.8	-3.3	0.5	1.0	4.6									
R	1.2	-1.4	-0.9	-1.1	0.4	-0.9	1.6	2.1	1.4	0.3	-1.6	2.1								
K	-1.0	-2.1	-2.4	-2.4	1.5	-1.1	-0.4	-1.1	0.1	-0.1	-0.3	-0.4	4.1							
M	-1.3	-3.5	-2.9	-2.7	-1.3	0.3	0.3	-2.4	1.6	-1.3	1.5	-0.6	3.0	4.7						
I	-2.4	1.1	-2.5	-2.3	-2.1	-0.3	-2.3	-0.9	-0.9	-2.0	1.1	-1.9	1.6	0.9	6.2					
L	-2.2	-1.2	-3.3	-3.0	-0.3	0.9	-1.5	-1.3	1.3	-1.9	-0.0	-0.9	1.8	0.3	3.0	5.8				
V	-5.2	-3.9	-5.1	-4.9	3.0	-4.9	-5.4	-5.4	-5.1	-1.8	-4.4	-4.0	1.9	-2.3	-3.2	-3.0	15.7			
F	-0.5	1.1	2.4	3.4	-2.8	-4.0	0.5	-2.5	-1.9	-2.7	-3.4	-1.6	-1.8	-0.9	-2.4	-3.2	-5.8	7.8		
Y	-3.6	-1.3	-3.8	-3.2	3.4	-0.2	-3.0	-3.0	-0.2	-3.2	-2.2	-1.4	-1.7	-3.3	-1.7	3.6	-3.0	-4.3	8.9	
W	-2.9	2.9	-1.4	-0.5	-0.1	-4.3	-2.6	-1.5	-2.4	-4.0	-5.2	-0.5	-3.3	-4.9	-1.9	-0.8	-3.4	-1.7	4.3	7.6
	C	S	T	P	A	G	N	D	E	Q	H	R	K	M	I	L	V	F	Y	W

Leaf-Leaf Medium Branches – Dayhoff Matrix

C	2.4																			
S	-1.5	4.8																		
T	1.1	1.9	3.7																	
P	0.3	2.5	3.7	4.5																
A	-1.2	-3.5	-2.5	-3.3	13.8															
G	0.0	-5.1	-3.1	-4.2	-4.0	5.1														
N	1.5	-0.9	1.1	1.1	-1.3	-1.2	2.8													
D	0.8	-0.8	-1.2	-1.8	-3.2	-1.6	0.5	5.7												
E	0.2	-3.7	-2.1	-2.6	-2.0	1.6	0.7	-0.6	3.6											
Q	1.3	-4.4	-1.8	-3.0	-1.9	1.0	0.0	-0.9	1.2	5.5										
H	-0.4	-4.8	-3.1	-4.1	-4.7	4.4	-1.6	-1.3	0.4	0.2	5.1									
R	1.3	-2.5	-1.0	-1.5	-0.0	0.1	1.5	0.8	1.9	2.0	-0.8	2.4								
K	-1.8	-3.2	-3.5	-3.7	-2.4	-1.7	-1.2	0.1	0.3	-1.1	-0.9	-0.8	4.6							
M	-1.9	-4.1	-3.6	-3.7	-4.4	-0.8	-1.0	-0.9	1.2	-1.6	0.1	-0.7	3.9	5.1						
I	-1.1	-2.2	-2.9	-3.2	-3.7	0.5	-1.1	0.5	0.1	-1.3	1.6	-0.7	2.3	2.2	3.6					
L	-2.0	-1.7	-3.5	-3.6	-2.5	-0.9	-1.6	0.4	0.7	-1.6	-0.7	-0.8	2.3	1.0	2.8	6.0				
V	-5.2	-2.5	-4.6	-4.9	-0.1	-6.2	-4.3	-4.0	-3.6	-3.6	-5.5	-3.4	3.4	-0.6	-1.2	-0.9	16.0			
F	-0.2	3.0	2.6	2.9	-3.1	-3.7	0.8	-1.2	-2.2	-2.7	-3.5	-1.3	-1.9	-2.2	-1.7	-2.1	-3.2	3.7		
Y	-3.3	-1.4	-3.7	-3.4	-0.4	-3.4	-3.2	-3.6	-1.7	-4.1	-4.4	-1.5	-2.9	-4.6	-1.9	2.5	-1.0	-2.6	9.7	
W	-3.5	2.0	-1.3	-0.8	-0.9	-5.6	-3.2	-3.4	-3.8	-5.3	-6.0	-2.7	-4.9	-6.3	-3.9	-0.8	-0.1	-0.2	6.4	8.7
	C	S	T	P	A	G	N	D	E	Q	H	R	K	M	I	L	V	F	Y	W

Leaf-Leaf Long Branches – Dayhoff Matrix

C	2.5																			
S	-1.7	4.2																		
T	-0.4	1.8	3.5																	
P	-1.2	2.7	3.3	4.1																
A	1.4	-0.5	0.8	-0.1	8.3															
G	-0.4	-3.6	-3.0	-3.7	-2.0	4.7														
N	0.5	-1.2	0.1	-0.5	0.8	-0.3	3.2													
D	0.3	-2.5	-2.2	-2.7	-1.7	-0.9	-0.2	8.1												
E	-0.2	-2.8	-2.3	-2.8	-0.5	2.0	0.3	-0.8	3.4											
Q	0.2	-4.6	-3.6	-4.7	-0.8	-0.2	-1.3	-1.6	0.5	6.6										
H	-0.1	-2.6	-2.0	-2.6	-1.8	2.6	-0.2	-0.6	0.9	-0.9	3.2									
R	1.4	-2.4	-1.4	-2.1	1.2	0.3	1.4	0.2	0.8	0.3	0.2	2.5								
K	-0.9	-2.2	-2.0	-2.5	-1.6	-0.5	-0.5	-1.4	0.2	-1.7	0.3	-0.4	5.8							
M	0.0	-2.0	-1.5	-2.1	-1.3	0.6	0.1	-0.3	0.7	-0.7	1.1	0.3	2.3	2.1						
I	0.0	-1.6	-1.5	-1.9	-1.3	1.0	0.1	-0.7	0.7	-1.1	1.6	0.3	1.2	1.3	2.8					
L	-0.7	-1.5	-1.9	-2.0	-1.2	0.1	-0.5	-1.4	1.5	-1.2	0.2	-0.2	0.6	0.6	0.7	5.9				
V	-3.5	-0.8	-2.9	-2.7	-2.7	-3.9	-3.5	-4.4	-2.2	-4.5	-3.7	-3.1	-2.7	-3.0	-2.5	-0.2	15.4			
F	-1.1	2.9	1.5	2.3	-0.2	-3.0	-0.7	-2.2	-2.1	-3.8	-2.1	-1.6	-1.6	-1.5	-1.1	-1.1	-0.9	5.3		
Y	-1.9	0.1	-1.2	-1.0	-1.1	-2.8	-1.9	-2.8	-1.3	-4.0	-2.3	-1.9	-1.6	-1.5	-1.5	2.5	4.3	-0.2	7.7	
W	-2.2	1.7	-0.1	0.5	-0.8	-3.9	-2.2	-3.0	-2.7	-4.8	-3.1	-2.5	-2.7	-2.6	-2.3	0.6	3.7	1.3	5.5	7.2
	C	S	T	P	A	G	N	D	E	Q	H	R	K	M	I	L	V	F	Y	W

Node-Node Short Branches (All Fractional) – Dayhoff Matrix

C	2.4																			
S	-2.3	5.4																		
T	-0.2	2.2	4.4																	
P	-1.2	3.1	4.3	5.0																
A	-0.3	-2.9	-0.7	-2.0	14.1															
G	0.0	-5.4	-3.7	-4.9	-3.9	4.6														
N	1.2	-1.8	-0.0	-0.4	-0.7	-0.4	3.3													
D	0.8	-3.1	-2.7	-3.7	-3.0	-1.1	-0.2	8.2												
E	0.2	-4.2	-3.0	-3.7	-2.4	2.4	0.7	-1.1	3.1											
Q	0.2	-6.6	-4.4	-6.0	-3.2	-0.2	-1.6	-2.5	0.0	7.6										
H	0.4	-4.2	-2.8	-3.8	-3.6	3.1	-0.1	-0.5	1.5	-0.9	3.0									
R	1.4	-3.1	-1.6	-2.5	0.4	0.5	1.7	0.8	1.3	0.3	0.4	2.2								
K	-0.4	-3.0	-3.0	-3.6	-2.0	-0.3	-0.3	-1.1	0.6	-1.7	0.7	-0.0	4.9							
M	0.0	-3.3	-2.6	-3.3	-3.1	0.9	0.2	-0.8	1.2	-1.6	1.6	0.3	3.1	3.1						
I	0.1	-2.6	-2.5	-3.1	-3.2	1.4	0.0	-0.4	1.1	-1.6	2.0	0.3	2.1	2.2	2.5					
L	-1.1	-2.9	-3.4	-3.8	-2.7	0.3	-0.8	-1.4	1.6	-2.6	0.3	-0.3	1.6	0.9	1.6	7.0				
V	-6.1	-2.4	-4.9	-4.7	-1.3	-7.1	-5.9	-7.2	-5.9	-5.6	-6.6	-4.9	-2.5	-5.1	-5.0	-2.1	16.7			
F	-1.3	3.6	2.2	2.9	-2.4	-3.8	-0.3	-2.9	-2.7	-5.0	-2.8	-1.8	-1.9	-1.9	-1.6	-2.4	-3.3	5.8		
Y	-4.0	-1.3	-3.1	-2.7	-1.0	-4.0	-3.4	-5.0	-2.1	-6.4	-4.2	-2.8	-2.8	-3.6	-2.8	3.6	3.0	-2.0	9.9	
W	-4.4	1.7	-1.1	-0.2	-2.2	-6.5	-3.9	-5.5	-4.5	-7.8	-6.0	-3.7	-4.8	-5.4	-4.6	0.0	2.2	0.3	6.5	9.4
	C	S	T	P	A	G	N	D	E	Q	H	R	K	M	I	L	V	F	Y	W

Node-Node Medium Branches (All Fractional) – Dayhoff Matrix

C	2.5																			
S	-1.9	4.7																		
T	-0.2	2.0	4.0																	
P	-1.3	2.9	3.8	4.7																
A	0.7	-1.2	0.4	-0.8	13.3															
G	-0.2	-4.5	-3.5	-4.6	-3.0	4.5														
N	0.8	-1.5	-0.0	-0.5	0.0	-0.3	3.2													
D	0.3	-3.1	-2.5	-3.5	-2.6	-0.7	-0.4	7.9												
E	-0.0	-3.5	-2.7	-3.4	-1.6	2.3	0.6	-0.9	3.1											
Q	0.1	-5.8	-4.4	-5.8	-2.2	-0.3	-1.6	-2.3	0.2	7.5										
H	0.2	-3.5	-2.5	-3.5	-2.7	2.7	-0.0	-0.3	1.3	-1.1	2.9									
R	1.2	-2.6	-1.5	-2.4	0.6	0.5	1.6	0.5	1.1	0.2	0.4	2.2								
K	-0.5	-2.3	-2.5	-2.9	-1.8	-0.4	-0.2	-0.9	0.5	-1.8	0.6	0.1	4.6							
M	-0.1	-2.6	-2.2	-2.8	-2.3	0.7	0.3	-0.5	1.1	-1.5	1.4	0.4	2.7	2.8						
I	0.0	-2.1	-2.0	-2.6	-2.1	1.1	0.1	-0.4	0.9	-1.5	1.8	0.3	1.8	1.8	2.2					
L	-1.0	-2.1	-2.7	-2.9	-1.7	0.3	-0.5	-1.2	1.5	-2.1	0.2	-0.1	1.3	0.8	1.3	6.5				
V	-3.9	-0.7	-2.7	-2.3	-1.8	-4.9	-3.8	-4.8	-3.9	-5.2	-4.5	-3.1	-1.8	-3.6	-3.2	-0.7	15.4			
F	-1.1	3.2	1.7	2.5	-0.9	-3.3	-0.4	-2.7	-2.3	-4.5	-2.3	-1.6	-1.5	-1.5	-1.2	-1.7	-1.2	5.3		
Y	-2.8	-0.3	-1.8	-1.3	-0.9	-3.2	-2.4	-3.6	-1.5	-5.1	-3.2	-2.0	-2.0	-2.6	-2.1	2.9	4.4	-0.7	9.0	
W	-3.1	2.0	-0.2	0.7	-1.1	-5.2	-2.8	-4.3	-3.5	-6.3	-4.6	-2.9	-3.5	-3.9	-3.3	0.1	3.5	1.1	5.9	8.4
	C	S	T	P	A	G	N	D	E	Q	H	R	K	M	I	L	V	F	Y	W

Node-Node Long Branches (All Fractional) – Dayhoff Matrix

C	2.5																			
S	-1.3	4.0																		
T	-0.0	1.6	3.6																	
P	-1.0	2.5	3.2	4.2																
A	1.0	-0.5	0.8	-0.3	11.3															
G	-0.5	-3.6	-3.0	-3.7	-2.5	4.5														
N	0.5	-1.1	-0.0	-0.5	0.2	-0.3	3.1													
D	0.1	-2.3	-1.9	-2.7	-1.9	-0.5	-0.4	7.3												
E	-0.4	-2.7	-2.3	-2.7	-1.3	2.1	0.4	-0.8	3.4											
Q	0.3	-4.4	-3.5	-4.6	-1.4	-0.1	-1.3	-1.5	0.3	6.9										
H	-0.0	-2.6	-2.1	-2.7	-2.1	2.4	-0.1	-0.2	0.9	-0.8	2.8									
R	0.9	-2.1	-1.3	-2.0	0.5	0.4	1.4	0.3	1.0	0.2	0.2	2.3								
K	-0.6	-1.8	-2.0	-2.3	-1.5	-0.4	-0.3	-0.7	0.3	-1.3	0.5	-0.0	4.3							
M	-0.3	-2.0	-1.9	-2.2	-1.8	0.5	0.1	-0.4	0.9	-1.1	1.2	0.2	2.3	2.7						
I	-0.2	-1.5	-1.7	-2.0	-1.7	0.9	-0.0	-0.4	0.7	-1.2	1.6	0.2	1.4	1.5	2.4					
L	-1.0	-1.6	-2.1	-2.3	-1.3	0.2	-0.5	-0.9	1.3	-1.6	0.1	-0.2	0.9	0.5	1.2	6.5				
V	-2.5	0.0	-1.5	-1.2	-1.0	-3.5	-2.4	-3.2	-2.7	-3.5	-3.0	-2.1	-1.0	-2.4	-2.0	-0.2	13.6			
F	-0.7	2.5	1.3	2.0	-0.3	-2.6	-0.3	-2.0	-1.8	-3.3	-1.8	-1.2	-1.1	-1.1	-0.8	-1.2	-0.4	4.8		
Y	-1.9	-0.0	-1.0	-0.7	-0.5	-2.4	-1.6	-2.6	-1.1	-3.6	-2.2	-1.4	-1.4	-1.8	-1.4	2.3	4.0	-0.3	7.7	
W	-2.0	1.9	0.2	0.9	-0.4	-3.8	-1.8	-3.0	-2.6	-4.5	-3.2	-2.1	-2.5	-2.8	-2.3	-0.0	3.3	1.1	4.7	7.0
	C	S	T	P	A	G	N	D	E	Q	H	R	K	M	I	L	V	F	Y	W

Node-Node Short Branches (Best Fractional) – Dayhoff Matrix

	C	S	T	P	A	G	N	D	E	Q	H	R	K	M	I	L	V	F	Y	W
C	2.3																			
S	-1.6	4.6																		
T	0.4	1.6	3.6																	
P	-0.5	2.4	3.5	4.3																
A	0.3	-1.4	-0.1	-0.8	9.5															
G	-0.5	-4.6	-2.9	-3.9	-2.1	4.7														
N	1.3	-1.1	0.3	0.2	0.3	-1.0	2.9													
D	0.9	-1.0	-1.4	-2.1	-0.8	-1.5	0.3	6.6												
E	-0.1	-3.1	-2.2	-2.5	-0.6	2.1	0.6	-0.8	3.2											
Q	0.8	-4.2	-2.2	-3.5	-0.9	0.7	-0.7	-1.2	0.3	5.5										
H	-0.3	-3.8	-2.3	-3.3	-2.5	3.5	-1.0	-1.3	0.8	0.2	4.2									
R	1.2	-1.9	-1.0	-1.5	1.4	-0.1	1.4	1.1	1.2	0.8	-0.6	2.0								
K	-0.9	-2.2	-2.5	-2.9	-0.0	-1.2	-0.9	-0.8	0.0	-0.5	-0.4	-0.5	4.9							
M	-0.9	-2.7	-2.4	-2.7	-1.8	0.1	-0.4	-1.1	1.0	-1.1	1.0	-0.4	3.2	4.2						
I	-0.9	-1.6	-2.3	-2.7	-2.0	0.5	-0.9	-0.2	0.3	-1.3	1.7	-0.7	2.0	2.1	4.3					
L	-1.6	-1.9	-2.9	-3.0	-1.0	0.1	-1.1	-0.9	1.3	-1.8	-0.2	-0.6	1.8	0.7	2.3	5.9				
V	-4.1	-1.0	-3.4	-3.2	1.5	-4.9	-3.9	-4.5	-3.9	-2.3	-4.9	-2.6	0.2	-2.9	-3.0	-0.9	14.8			
F	-0.9	2.9	2.0	2.7	-1.3	-3.7	0.2	-1.7	-2.1	-3.2	-3.1	-1.4	-1.7	-1.6	-1.3	-2.1	-2.4	5.5		
Y	-3.0	-0.6	-2.5	-2.0	0.9	-2.5	-2.3	-3.3	-1.1	-4.0	-3.5	-1.5	-1.7	-3.0	-1.6	3.6	2.7	-1.7	8.4	
W	-2.9	1.9	-0.7	0.1	-0.2	-4.6	-2.4	-3.1	-2.6	-4.7	-4.9	-1.6	-3.1	-4.2	-3.1	0.7	2.7	0.3	5.7	7.7

Node-Node Medium Branches (Best Fractional) – Dayhoff Matrix

	C	S	T	P	A	G	N	D	E	Q	H	R	K	M	I	L	V	F	Y	W
C	2.2																			
S	-1.5	4.0																		
T	0.0	1.7	3.4																	
P	-1.0	2.7	3.2	4.2																
A	1.0	-0.6	0.8	-0.3	9.8															
G	-0.3	-4.1	-3.0	-4.1	-2.0	4.8														
N	0.6	-1.1	0.3	-0.2	0.5	-0.4	3.1													
D	0.4	-2.5	-1.8	-2.8	-1.6	-0.5	-0.4	7.0												
E	-0.2	-3.0	-2.3	-2.9	-0.8	2.3	0.5	-1.0	3.6											
Q	0.5	-4.8	-3.4	-4.8	-1.2	0.2	-1.3	-1.7	0.7	6.3										
H	0.2	-2.9	-1.9	-2.9	-1.7	2.7	-0.1	-0.1	0.9	-0.7	3.2									
R	1.1	-2.2	-1.1	-2.0	1.0	0.5	1.5	0.5	1.1	0.5	0.2	2.3								
K	-0.7	-1.9	-2.0	-2.5	-1.1	-0.9	-0.3	-0.9	0.3	-1.5	0.2	-0.1	5.0							
M	-0.2	-2.1	-1.7	-2.3	-1.4	0.4	0.2	-0.4	0.9	-1.1	1.2	0.2	2.8	3.1						
I	-0.1	-1.4	-1.5	-2.0	-1.3	0.8	0.0	-0.3	0.7	-1.1	1.7	0.1	1.6	1.7	2.5					
L	-1.1	-1.7	-2.2	-2.5	-1.1	0.2	-0.7	-1.2	1.5	-1.6	-0.0	-0.3	1.3	0.6	1.4	6.3				
V	-3.3	0.0	-2.0	-1.5	-1.2	-4.4	-3.2	-4.1	-3.3	-4.4	-3.8	-2.6	-1.3	-3.1	-2.5	0.1	13.5			
F	-0.9	2.8	1.4	2.3	-0.4	-3.1	-0.3	-2.3	-2.2	-3.7	-2.0	-1.4	-1.3	-1.3	-0.8	-1.5	-0.6	4.8		
Y	-2.4	0.1	-1.3	-0.8	-0.5	-2.9	-2.0	-3.1	-1.2	-4.3	-2.8	-1.8	-1.6	-2.3	-1.6	2.8	4.5	-0.3	7.6	
W	-2.5	2.0	0.1	1.0	-0.6	-4.5	-2.2	-3.5	-2.9	-5.2	-3.8	-2.5	-2.8	-3.2	-2.5	0.5	3.8	1.3	5.3	6.8

Node-Node Long Branches (Best Fractional) – Dayhoff Matrix

	C	S	T	P	A	G	N	D	E	Q	H	R	K	M	I	L	V	F	Y	W
C	2.3																			
S	-1.0	3.5																		
T	0.1	1.5	3.2																	
P	-0.8	2.4	2.9	3.8																
A	1.1	-0.3	0.9	-0.2	9.7															
G	-0.5	-3.4	-2.8	-3.6	-2.2	4.8														
N	0.4	-1.0	0.1	-0.3	0.4	-0.3	3.2													
D	0.1	-2.1	-1.6	-2.3	-1.5	-0.3	-0.3	6.5												
E	-0.4	-2.6	-2.2	-2.7	-1.1	2.2	0.4	-0.7	3.9											
Q	0.3	-4.0	-3.1	-4.3	-1.2	0.0	-1.3	-1.4	0.5	6.1										
H	-0.0	-2.3	-1.8	-2.5	-1.8	2.4	-0.0	-0.1	0.9	-0.7	2.9									
R	0.9	-1.9	-1.2	-1.9	0.6	0.5	1.4	0.4	1.0	0.3	0.3	2.4								
K	-0.6	-1.6	-1.8	-2.0	-1.3	-0.5	-0.2	-0.6	0.3	-1.2	0.5	-0.0	4.4							
M	-0.3	-1.8	-1.6	-2.0	-1.5	0.5	0.2	-0.2	0.9	-0.9	1.3	0.2	2.3	2.8						
I	-0.2	-1.3	-1.4	-1.8	-1.4	0.8	-0.0	-0.3	0.7	-1.0	1.6	0.2	1.4	1.5	2.8					
L	-1.0	-1.5	-1.9	-2.1	-1.2	0.2	-0.6	-0.9	1.3	-1.4	0.1	-0.2	0.9	0.4	1.2	6.7				
V	-2.3	0.2	-1.3	-1.0	-1.0	-3.4	-2.3	-3.0	-2.6	-3.4	-2.8	-2.1	-0.9	-2.3	-1.9	-0.1	12.5			
F	-0.6	2.3	1.2	1.9	-0.3	-2.5	-0.3	-1.8	-1.8	-3.1	-1.6	-1.2	-1.0	-1.0	-0.6	-1.1	-0.3	4.5		
Y	-1.7	0.1	-0.9	-0.5	-0.4	-2.3	-1.5	-2.3	-1.1	-3.4	-2.0	-1.4	-1.2	-1.7	-1.2	2.3	3.9	-0.2	6.9	
W	-1.8	1.8	0.3	1.0	-0.3	-3.6	-1.7	-2.7	-2.5	-4.1	-2.9	-2.0	-2.2	-2.5	-2.0	0.1	3.2	1.2	4.3	6.1

Node-Node Short Branches (Best Count) – Dayhoff Matrix

C	2.2																			
S	-1.6	4.7																		
T	0.3	1.7	3.7																	
P	-0.6	2.5	3.6	4.4																
A	0.3	-1.5	-0.1	-1.0	10.5															
G	-0.3	-4.5	-2.9	-3.9	-2.3	4.6														
N	1.2	-1.2	0.2	0.1	0.2	-0.8	2.8													
D	0.8	-1.4	-1.6	-2.3	-1.0	-1.3	0.1	7.0												
E	-0.1	-3.2	-2.3	-2.6	-0.9	2.1	0.6	-0.9	3.1											
Q	0.7	-4.4	-2.6	-3.9	-1.2	0.6	-0.8	-1.4	0.4	5.9										
H	-0.1	-3.6	-2.2	-3.2	-2.5	3.3	-0.7	-1.0	0.9	-0.0	3.8									
R	1.2	-2.1	-1.1	-1.7	1.2	0.1	1.4	1.0	1.2	0.8	-0.4	2.0								
K	-0.8	-2.2	-2.4	-2.8	-0.3	-1.2	-0.7	-0.8	0.0	-0.7	-0.3	-0.4	4.9							
M	-0.6	-2.6	-2.2	-2.6	-1.9	0.2	-0.2	-0.9	0.9	-1.0	1.1	-0.3	3.0	3.8						
I	-0.5	-1.6	-2.0	-2.5	-2.0	0.6	-0.6	-0.2	0.4	-1.2	1.7	-0.5	1.8	1.9	3.8					
L	-1.4	-2.0	-2.9	-3.0	-1.3	0.1	-1.0	-1.0	1.3	-1.8	-0.2	-0.5	1.7	0.6	2.0	6.1				
V	-4.3	-1.1	-3.5	-3.3	1.1	-5.4	-4.2	-4.8	-4.3	-2.7	-5.1	-2.9	-0.4	-3.4	-3.3	-1.1	15.3			
F	-0.9	2.9	1.9	2.6	-1.4	-3.6	0.1	-1.9	-2.2	-3.3	-2.8	-1.4	-1.5	-1.4	-1.2	-2.1	-2.4	5.5		
Y	-3.0	-0.7	-2.4	-2.0	0.6	-2.7	-2.3	-3.5	-1.1	-4.2	-3.4	-1.6	-1.8	-2.9	-1.8	3.6	2.9	-1.7	8.6	
W	-3.0	1.8	-0.7	0.1	-0.5	-4.8	-2.5	-3.4	-2.9	-5.0	-4.9	-1.9	-3.3	-4.1	-3.3	0.6	2.8	0.3	5.8	8.0
	C	S	T	P	A	G	N	D	E	Q	H	R	K	M	I	L	V	F	Y	W

Node-Node Medium Branches (Best Count) – Dayhoff Matrix

C	2.2																			
S	-1.5	4.1																		
T	-0.1	1.6	3.6																	
P	-1.2	2.7	3.3	4.5																
A	0.9	-0.6	0.8	-0.4	11.1															
G	-0.3	-3.9	-3.0	-4.2	-2.3	4.6														
N	0.5	-1.1	0.1	-0.3	0.2	-0.4	3.0													
D	0.3	-2.3	-1.9	-2.9	-1.8	-0.4	-0.3	7.0												
E	-0.2	-2.9	-2.3	-3.0	-1.1	2.1	0.4	-0.9	3.3											
Q	0.3	-4.6	-3.5	-4.9	-1.4	0.2	-1.4	-1.6	0.6	6.7										
H	0.2	-2.8	-2.0	-3.0	-1.9	2.4	-0.1	-0.1	0.8	-0.8	2.9									
R	0.9	-2.2	-1.3	-2.2	0.8	0.5	1.3	0.5	1.0	0.4	0.2	2.2								
K	-0.5	-1.7	-2.0	-2.5	-1.2	-0.8	-0.3	-0.7	0.2	-1.4	0.2	-0.1	4.5							
M	-0.1	-2.1	-1.8	-2.4	-1.6	0.3	0.2	-0.4	0.9	-1.1	1.1	0.2	2.4	2.8						
I	-0.0	-1.4	-1.6	-2.1	-1.5	0.7	0.0	-0.2	0.7	-1.2	1.5	0.2	1.4	1.5	2.3					
L	-0.9	-1.6	-2.2	-2.5	-1.2	0.2	-0.5	-1.0	1.4	-1.6	0.0	-0.1	1.2	0.5	1.2	6.1				
V	-3.0	-0.0	-1.9	-1.6	-0.9	-4.1	-3.0	-3.9	-3.1	-4.0	-3.5	-2.4	-1.1	-3.0	-2.5	-0.2	14.2			
F	-0.8	2.7	1.4	2.2	-0.5	-3.0	-0.3	-2.2	-2.0	-3.5	-1.9	-1.4	-1.2	-1.2	-0.8	-1.4	-0.6	5.1		
Y	-2.1	0.0	-1.2	-0.8	-0.4	-2.7	-1.8	-2.8	-1.2	-4.0	-2.6	-1.5	-1.4	-2.1	-1.6	2.7	4.4	-0.4	8.0	
W	-2.4	2.0	0.1	1.0	-0.4	-4.4	-2.1	-3.3	-2.9	-5.1	-3.7	-2.3	-2.7	-3.2	-2.5	0.3	3.7	1.2	5.3	7.4
	C	S	T	P	A	G	N	D	E	Q	H	R	K	M	I	L	V	F	Y	W

Node-Node Long Branches (Best Count) – Dayhoff Matrix

C	2.4																			
S	-1.1	3.7																		
T	0.0	1.5	3.4																	
P	-0.9	2.3	3.0	4.2																
A	1.0	-0.3	0.9	-0.3	10.2															
G	-0.5	-3.3	-2.8	-3.6	-2.2	4.5														
N	0.3	-1.0	0.1	-0.4	0.3	-0.3	3.1													
D	0.1	-2.0	-1.7	-2.4	-1.5	-0.3	-0.3	6.6												
E	-0.5	-2.5	-2.2	-2.6	-1.2	2.0	0.4	-0.7	3.7											
Q	0.3	-3.9	-3.1	-4.3	-1.2	0.1	-1.3	-1.2	0.4	6.4										
H	-0.0	-2.3	-1.8	-2.5	-1.8	2.1	-0.1	-0.1	0.8	-0.7	2.7									
R	0.8	-1.9	-1.3	-2.0	0.5	0.5	1.2	0.3	0.9	0.3	0.2	2.4								
K	-0.5	-1.5	-1.8	-2.1	-1.2	-0.5	-0.3	-0.6	0.2	-1.1	0.4	-0.0	4.1							
M	-0.3	-1.7	-1.6	-2.0	-1.5	0.4	0.1	-0.3	0.8	-0.9	1.1	0.2	2.0	2.6						
I	-0.2	-1.2	-1.5	-1.9	-1.4	0.6	-0.1	-0.3	0.6	-1.0	1.5	0.1	1.3	1.3	2.6					
L	-0.9	-1.4	-1.9	-2.1	-1.1	0.2	-0.5	-0.8	1.2	-1.3	0.0	-0.2	0.9	0.4	1.1	6.3				
V	-2.2	0.3	-1.2	-1.0	-0.8	-3.1	-2.2	-2.7	-2.5	-3.0	-2.6	-1.9	-0.7	-2.2	-1.8	-0.1	12.9			
F	-0.6	2.3	1.2	1.9	-0.3	-2.5	-0.2	-1.7	-1.7	-2.9	-1.6	-1.2	-1.0	-1.0	-0.6	-1.1	-0.3	4.8		
Y	-1.6	0.1	-0.8	-0.5	-0.2	-2.1	-1.4	-2.1	-0.9	-3.1	-1.9	-1.2	-1.1	-1.6	-1.2	2.2	3.9	-0.2	7.1	
W	-1.7	1.8	0.3	1.0	-0.2	-3.5	-1.6	-2.5	-2.4	-3.9	-2.9	-1.9	-2.1	-2.5	-2.0	0.0	3.3	1.2	4.3	6.6
	C	S	T	P	A	G	N	D	E	Q	H	R	K	M	I	L	V	F	Y	W
