# Supplementary material for: Integrating protein structures and precomputed genealogies in the Magnum database: Examples with cellular retinoid binding proteins
Source: BMC Bioinformatics. 2006 Feb 23;7:89. doi: 10.1186/1471-2105-7-89 (PMC1475641; doi:10.1186/1471-2105-7-89)
Supplement: Additional File 2 — F-to-E/Q/K replacements. List of F-to-E/Q/K replacements on short branches and at buried sites. [file 1471-2105-7-89-S2.pdf]

Dataset S2. F-to-E/Q/K replacements on short branches and at buried sites

ij: parent node (i) and child node (j) residues.
family and description: PirSF identifier and family description
members, length, chains: The number of homologs, the alignment length, and the number of PDB chains associated with a family
site: alignment site of the ij replacement
parent_node, child_node: branch on which the ij event occurs
child_name: an approximate description of the leaf sequences below the child node.
bl: branch length
bf_bc: changes along the branch counted by the best fractional method
pprob, cpprob: posterior probability values of the ij residues
alpha: alignment based substitution rate factor
SA, SS: solvent accessible surface area, and secondary structure type of the alignment site
aai, aaj: frequencies of the i and j residues in the leaf sequences at this alignment site
sum_ints: frequency of all intermediate residues between i and j in the leaf sequences.

ij	family	description	members	length	chains	site	parent	child	child name	bl	bf bc	pprob	cprob	alpha	SA	SS	aai	aaj	ints
EF	SF000076	hydroxylamine reductase	48	624	3	211	62	65	cellularorg	.1015	16.17	0.65	0.99	1.09	5	H	0.21	0.52	0.02
FK	SF000080	plastocyanin/Azurin	127	140	48	19	224	NF01327006	Burkholder:	.0328	4.96	1.00	1.00	.23	17	H	0.83	0.01	0.10
EF	SF000090	electron transfer flavoprote	168	352	3	215	291	292	Actinobacte	.0978	10.10	0.78	0.99	.83	10	B	0.58	0.04	0.04
FE	SF000090	electron transfer flavoprote	168	352	3	133	230	231	Proteobacte	.0365	12.02	0.81	0.74	1.64	27	H	0.06	0.40	0.13
KF	SF000117	ketol-acid reductoisomerase,	121	374	1	164	193	197	cellularorg	.0473	14.23	0.47	0.55	1.66	21	C	0.12	0.11	0.07
KF	SF000121	D-3-phosphoglycerate dehydro	130	600	1	30	137	138	Bacteria	.0765	8.28	0.65	0.80	.64	28	T	0.05	0.07	0.05
FE	SF000127	xanthine dehydrogenase	80	1553	2	483	128	129	Poaceae	.0815	46.18	0.58	0.69	.44	5	H	0.72	0.09	0.08
EF	SF000129	carbon monoxide dehydrogenas	122	929	2	108	136	141	Thermoprote	.1052	17.25	0.56	0.56	1.00	30	T	0.10	0.04	0.24
QF	SF000153	Escherichia coli arsenate re	31	141	3	120	54	55	Rhizobiacea	.0773	7.96	0.61	0.50	3.30	26	C	0.52	0.10	0.10
FQ	SF000154	isopenicillin N synthase	74	421	1	69	96	97	cellularorg	.0908	7.40	0.34	0.32	1.69	2	H	0.08	0.05	0.22
KF	SF000167	oxygen-independent coproporp	77	499	1	211	148	149	Gammaprote	.0113	3.63	0.75	0.96	1.81	21	H	0.18	0.12	0.33
FQ	SF000168	acyl-CoA oxidase	96	842	1	422	122	128	Caenorhabd:	.0887	21.47	0.31	0.31	1.01	16	H	0.05	0.14	0.59
FQ	SF000168	acyl-CoA oxidase	96	842	1	144	106	107	cellularorg	.0967	34.37	1.00	0.65	1.34	22	C	0.06	0.09	0.33
FK	SF000168	acyl-CoA oxidase	96	842	1	831	147	NF01174395	Musmusculus	.0532	34.00	1.00	1.00	.38	14	H	0.22	0.01	0.62
EF	SF000168	acyl-CoA oxidase	96	842	1	718	175	178	Saccharomyc	.0134	6.09	0.29	0.87	1.94	8	C	0.06	0.09	0.26
FQ	SF000178	succinate dehydrogenase cytc	73	143	1	142	97	NF00479148	Marchantia	.1197	7.25	0.67	1.00	1.26	17	H	0.07	0.02	0.15
FE	SF000182	acyl-CoA dehydrogenase	184	533	4	55	292	293	Proteobacte	.0567	7.73	0.93	0.46	.77	24	C	0.45	0.02	0.36
FQ	SF000186	glutamate synthase large sub	125	1874	2	767	197	198	Alphaprote	.0527	34.38	0.66	0.94	1.10	4	H	0.28	0.06	0.46
FQ	SF000186	glutamate synthase large sub	125	1874	2	288	233	247	Bacteria	.0257	22.24	0.68	0.37	2.50	30	C	0.11	0.02	0.27
QF	SF000190	pyridoxamine-phosphate oxida	95	304	4	68	169	170	cellularorg	.1176	5.88	0.91	0.55	.97	1	H	0.40	0.02	0.38
FQ	SF000244	sulfite/ferredoxin-nitrite r	142	682	1	487	202	NF00260830	Zeamays	.0584	23.10	0.82	1.00	.55	7	T	0.18	0.01	0.57
FK	SF000255	5,10-methylenetetrahydrofola	64	306	2	221	82	83	Bacteria	.1152	9.67	0.99	0.91	.63	23	H	0.59	0.05	0.16
FQ	SF000256	nitrous-oxide reductase	18	619	1	81	28	NF00576750	Achromobac	.0670	30.50	0.99	1.00	1.00	2	H	0.88	0.12	0.00
QF	SF000264	methyl coenzyme M reductase,	31	262	4	96	46	48	environmen	.0947	19.57	0.99	1.00	.64	7	T	0.13	0.16	0.71
FK	SF000264	methyl coenzyme M reductase,	31	262	4	252	43	NF00186564	Methanosarc	.0805	16.69	0.80	1.00	.74	4	H	0.03	0.03	0.19
FK	SF000294	Pseudomonas cytochrome-c per	28	382	3	277	43	44	Proteobacte	.1083	9.94	0.56	0.85	1.02	18	H	0.45	0.20	0.15
FQ	SF000306	hydrogenase (NiFe) large cha	59	712	7	574	60	100	Proteobacte	.0156	4.61	0.99	1.00	1.69	2	H	0.31	0.12	0.29
FK	SF000315	lipoxygenase	98	990	7	87	188	NF01250121	Nicotianaat	.0396	34.30	0.99	1.00	.98	15	H	0.50	0.02	0.31
FK	SF000328	toluene dioxygenase ferredox	100	488	2	408	134	136	Actinobacte	.0638	8.44	0.61	0.34	1.04	1	B	0.45	0.06	0.04
FQ	SF000328	toluene dioxygenase ferredox	100	488	2	321	151	184	Proteobacte	.0322	6.23	0.92	0.83	1.69	24	B	0.22	0.12	0.17
FE	SF000356	herpesvirus ribonucleoside-c	59	1178	7	776	62	63	Viridiplant	.0759	42.89	0.66	0.97	1.49	2	B	0.34	0.08	0.22
EF	SF000358	anaerobic ribonucleoside-tri	64	742	1	269	83	84	Bacteria	.1091	32.57	1.00	0.40	1.58	0	H	0.45	0.19	0.17
KF	SF000358	anaerobic ribonucleoside-tri	64	742	1	503	104	NF02019762	Bacteriopha	.0053	3.00	1.00	1.00	.61	0	H	0.02	0.62	0.33
KF	SF000358	anaerobic ribonucleoside-tri	64	742	1	545	77	80	root	.1164	45.89	1.00	0.98	1.26	12	C	0.30	0.40	0.24
FK	SF000361	ferredoxin--NADP reductase	54	396	30	271	61	62	Arabidopsis	.0691	20.45	1.00	1.00	.76	8	C	0.11	0.04	0.65
EF	SF000380	formate C-acetyltransferase	29	842	1	158	49	53	Deltaprote	.1039	10.78	0.16	0.12	1.57	5	H	0.45	0.07	0.13
FK	SF000388	3-methyl-2-oxobutanoate hydr	120	309	1	308	124	125	Archaea	.0747	14.90	1.00	0.79	.61	28	C	0.55	0.03	0.29
EF	SF000414	purH bifunctional enzyme	49	679	3	479	62	67	Bacillus	.0807	25.96	0.89	0.96	1.70	6	C	0.16	0.08	0.45
FQ	SF000426	arylamine N-acetyltransferas	68	375	2	72	124	128	Proteobacte	.1105	6.21	0.44	0.45	.81	21	H	0.59	0.04	0.09
FE	SF000428	phosphate acetyltransferase	107	381	2	13	164	165	Firmicutes	.0864	10.88	0.50	0.36	1.18	30	H	0.32	0.09	0.27
FE	SF000465	glycogenin (glycosyl transfe	25	407	1	321	27	30	root	.0969	23.74	1.00	0.99	1.00	19	T	0.30	0.30	0.00
FK	SF000502	spermidine synthase	99	359	3	280	102	106	Magnoliophy	.0722	11.13	1.00	1.00	.44	6	C	0.71	0.27	0.02
QF	SF000521	4-aminobutyrate transaminase	27	492	1	39	43	44	Ascomycota	.0802	10.94	0.42	0.63	2.05	22	T	0.56	0.19	0.11
FQ	SF000532	ATP-dependent phosphofructok	113	445	5	190	125	137	Pasteurella	.0589	20.03	0.94	1.00	.62	17	H	0.12	0.04	0.06
FE	SF000532	ATP-dependent phosphofructok	113	445	4	444	138	139	Pasteurella	.0134	3.70	0.82	0.67	1.94	14	T	0.21	0.01	0.12
KF	SF000535	6-phosphofructokinase 2	109	353	1	339	112	113	Bacilli	.0325	8.53	0.35	0.83	2.03	15	H	0.10	0.12	0.24
EF	SF000544	adenylylsulfate kinase	63	215	1	89	71	72	Bacteria	.1013	5.85	0.44	0.94	.58	28	H	0.81	0.06	0.07
FK	SF000601	Tyrosine-protein kinase, pro	131	797	4	147	257	NF00069377	Macacafasc:	.0392	43.87	0.91	1.00	3.19	24	T	0.03	0.03	0.13
FK	SF000601	Tyrosine-protein kinase, pro	131	797	2	781	225	226	Metazoa	.0393	8.79	0.37	0.75	1.26	25	H	0.45	0.09	0.04
FQ	SF000601	Tyrosine-protein kinase, pro	131	797	2	781	161	162	Roussarcoma	.0159	13.36	1.00	1.00	1.26	25	H	0.45	0.15	0.03
EF	SF000628	basic fibroblast growth fact	39	384	1	366	41	NF00050229	Gallusgallu	.0386	3.09	0.71	1.00	3.26	1	H	0.18	0.03	0.29
EF	SF000676	Homoserine kinase	159	421	1	417	244	245	Bacilli	.0562	11.23	0.98	0.99	.66	5	C	0.06	0.03	0.08
KF	SF000676	Homoserine kinase	159	421	1	138	225	226	Campylobac	.0510	6.34	0.82	0.90	1.56	29	C	0.37	0.05	0.31
KF	SF000699	phosphotransferase system, l	89	106	2	42	160	163	Lactobacil	.0605	4.71	0.36	0.25	1.09	7	H	0.29	0.03	0.43
FQ	SF000728	N-acetylglutamate kinase	131	301	1	144	154	155	Betaproteol	.0041	2.15	0.62	0.98	1.25	24	T	0.30	0.06	0.50
FQ	SF000728	N-acetylglutamate kinase	131	301	1	144	159	NF01974918	Burkholder:	.0916	22.85	0.70	1.00	1.25	24	T	0.30	0.06	0.50
FK	SF000730	creatine kinase	100	406	11	366	107	108	Eukaryota	.0267	8.96	0.99	0.94	1.58	24	T	0.21	0.13	0.40
KF	SF000766	transcription initiation fac	87	422	1	111	107	NF00585485	Burkholder:	.0339	7.34	0.45	1.00	2.19	19	T	0.07	0.01	0.20
EF	SF000796	DNA-directed DNA polymerase	60	1157	15	657	62	63	Bacteria	.0695	6.56	0.22	0.29	1.63	24	H	0.15	0.07	0.47
FE	SF000796	DNA-directed DNA polymerase	60	1157	15	605	72	NF00575240	Helicobacte	.0795	52.97	0.88	1.00	1.56	28	T	0.28	0.02	0.24
KF	SF000796	DNA-directed DNA polymerase	60	1157	7	624	72	NF00575240	Helicobacte	.0795	52.97	0.73	1.00	1.72	29	H	0.02	0.04	0.30
QF	SF000814	poly(A) polymerase/tRNA nucl	89	705	2	413	94	95	Pasteurella	.0729	15.54	0.39	0.92	1.96	27	H	0.04	0.06	0.16
FE	SF000814	poly(A) polymerase/tRNA nucl	89	705	2	245	105	155	Proteobacte	.0699	7.54	0.54	0.75	1.16	14	C	0.20	0.25	0.15
FQ	SF000868	14-3-3 protein	94	269	2	11	128	129	coreeudicot	.0509	10.86	0.93	0.97	1.84	22	H	0.12	0.02	0.46
EF	SF000871	cholinesterase	66	914	17	266	83	NF00039471	Muscadomes	.0998	33.64	0.85	1.00	.29	24	T	0.02	0.03	0.02
FE	SF000879	carboxymethylenebutenolidase	66	315	2	56	97	NF00292027	Caenorhabd:	.0077	1.91	0.70	1.00	1.16	15	H	0.21	0.02	0.21
KF	SF000891	Alkaline phosphatase	111	682	19	309	191	192	Eutheria	.0981	31.20	0.35	1.00	1.41	13	T	0.31	0.11	0.27
EF	SF000901	phosphoserine phosphatase	109	284	2	7	192	202	Pasteurella	.1175	15.52	0.28	1.00	2.12	22	T	0.05	0.10	0.19
FQ	SF000911	Serine/threonine protein pho	56	642	1	591	82	NF00499489	Musmusculus	.0937	41.58	1.00	1.00	.38	22	H	0.98	0.02	0.00
FK	SF000960	3',5'-cyclic-nucleotide phos	30	402	1	43	31	NF00162577	Bostaurus	.0169	5.00	1.00	1.00	2.39	13	H	0.17	0.03	0.00
FK	SF000989	deoxyribonuclease IV (phage	32	318	1	17	49	50	Schizosacch	.0476	13.00	1.00	1.00	2.32	14	H	0.03	0.09	0.28
KF	SF000989	deoxyribonuclease IV (phage	32	318	1	24	49	50	Schizosacch	.0476	13.00	1.00	1.00	2.40	28	H	0.06	0.09	0.21
FK	SF001017	tRNA nucleotidyltransferase	120	291	6	265	173	174	Actinobacte	.0137	3.31	0.99	0.41	1.81	1	H	0.21	0.02	0.36
FQ	SF001017	tRNA nucleotidyltransferase	120	291	6	192	154	166	cellularorg	.0378	3.16	0.62	0.21	2.26	9	C	0.16	0.07	0.40
FQ	SF001024	alpha-amylase, fungal type	28	525	3	13	30	35	Fungi	.1190	36.02	1.00	1.00	1.15	3	B	0.50	0.46	0.00
FE	SF001026	neopullulanase	69	798	13	156	130	131	Bacteria	.0685	7.86	0.22	0.35	.43	1	B	0.62	0.01	0.22
QF	SF001026	neopullulanase	69	798	13	641	80	82	cellularorg	.0861	28.74	0.99	0.83	1.04	7	T	0.17	0.30	0.35
FK	SF001028	alpha-amylase, plant type	46	418	2	210	76	79	rosids	.0162	7.19	0.55	0.92	1.90	3	C	0.07	0.13	0.62
KF	SF001061	Streptomyces chitinase chi40	20	465	1	211	33	NF00756606	Hypocreali:	.1004	35.64	0.75	1.00	2.39	1	H	0.15	0.05	0.25
FQ	SF001079	Agrobacterium beta-glucosida	151	742	15	446	167	168	Bacteria	.0778	8.98	0.77	0.89	.86	18	T	0.44	0.03	0.15
FE	SF001083	beta-galactosidase	68	1298	5	1147	123	124	Pasteurella	.0411	6.37	0.84	0.37	1.20	19	C	0.28	0.01	0.35
QF	SF001084	beta-galactosidase, BgaH typ	48	748	1	328	52	53	Bacilli	.0151	6.44	0.16	0.16	2.54	0	H	0.09	0.09	0.11
EF	SF001085	beta-fructofuranosidase	175	752	1	17	254	NF00656992	Arabidopsis	.0035	3.68	0.69	1.00	2.88	10	B	0.13	0.02	0.26
KF	SF001085	beta-fructofuranosidase	175	752	1	101	214	NF00305475	Schizosacch	.0889	39.00	1.00	1.00	.75	0	B	0.01	0.01	0.04

EF	SF001085	beta-fructofuranosidase	175	752	1	9	297	305	Solanum	.0157	7.62	0.38	0.94	2.58	18	C	0.07	0.09	0.38
FK	SF001122	human methionyl aminopeptida	33	507	3	507	60	NF01485107	Neurospora	.0431	19.00	1.00	1.00	.37	24	C	0.06	0.03	0.91
FK	SF001130	Folate hydrolase G	131	629	3	614	243	245	Streptococ	.0187	7.23	0.31	0.96	1.41	23	H	0.02	0.10	0.37
QF	SF001154	complement B/C2	28	347	1	313	39	40	Cyprinusca	.1099	8.25	0.56	1.00	1.26	5	C	0.19	0.08	0.00
FE	SF001195	thimet oligopeptidase	21	789	1	705	22	NF00568114	Rattusnorv	.0705	44.21	1.00	1.00	.20	16	C	0.95	0.05	0.00
EF	SF001201	zinc metalloendopeptidase, r	106	430	5	54	152	170	Bacilli	.0761	12.92	0.83	0.84	2.52	24	B	0.13	0.17	0.11
FE	SF001206	atrolysin C	33	205	9	80	64	NF00410088	Crotalusad	.0798	15.81	1.00	1.00	.38	0	H	0.97	0.03	0.00
FE	SF001207	trigramin precursor	27	211	2	6	41	42	Crotalinae	.0984	9.69	0.99	0.99	.63	5	T	0.77	0.14	0.00
EF	SF001208	signal peptidase I	27	338	2	153	40	41	Enterobact	.0821	5.61	0.18	0.37	2.60	30	B	0.07	0.19	0.08
FE	SF001212	multicatalytic endopeptidase	114	357	39	219	123	NF00048984	Gallusgall	.0617	11.60	0.99	1.00	1.29	26	H	0.29	0.11	0.39
FK	SF001237	dihydroorotase	57	435	1	105	104	107	Gammaprote	.0883	18.80	0.93	0.88	2.35	5	T	0.11	0.07	0.21
EF	SF001238	cyclic amidohydrolases (dihy	111	630	3	383	148	149	Bacteria	.1002	16.89	0.34	0.98	1.71	18	T	0.17	0.06	0.17
FQ	SF001249	adenosine deaminase	68	404	7	212	75	76	Vibrionace	.0920	21.58	0.99	1.00	1.32	4	T	0.33	0.06	0.28
EF	SF001290	vacuolar H(+)-transporting A	14	558	1	72	16	18	cellularor	.0467	5.03	0.49	0.97	.94	13	C	0.20	0.20	0.10
FQ	SF001320	DNA helicase recG	126	969	1	393	215	216	Bacillus	.1049	46.26	0.99	0.53	1.60	13	T	0.30	0.06	0.15
EF	SF001334	Escherichia coli glutamate c	70	553	1	122	116	117	Archaea	.0970	10.43	0.36	0.88	.76	20	H	0.67	0.03	0.09
FK	SF001335	ornithine decarboxylase	95	535	4	208	117	119	Bacteria	.1173	23.72	0.99	1.00	.37	28	T	0.66	0.07	0.22
FK	SF001337	diaminopimelate decarboxylas	143	502	2	260	146	147	Bacilli	.0707	22.43	0.70	0.69	.83	2	H	0.11	0.05	0.55
EF	SF001339	phosphoribosylaminoimidazole	155	462	4	244	225	226	cellularor	.0993	8.05	0.57	0.96	.74	19	B	0.45	0.03	0.03
EF	SF001347	phosphoenolpyruvate carboxyl	161	1259	2	71	298	299	Proteobact	.0534	20.33	0.51	1.00	1.15	11	H	0.05	0.05	0.59
KF	SF001348	phosphoenolpyruvate carboxyk	71	719	1	693	133	135	Actinobact	.1016	33.32	0.44	0.97	2.25	25	H	0.14	0.10	0.10
FK	SF001348	phosphoenolpyruvate carboxyk	71	719	1	690	87	NF01161134	Caenorhabd	.0258	16.39	1.00	1.00	.85	16	H	0.11	0.01	0.71
QF	SF001349	uroporphyrinogen-III decarbo	173	472	9	168	267	NF00172073	Chlamydiam	.0998	24.25	1.00	1.00	.43	18	T	0.03	0.01	0.68
FE	SF001349	uroporphyrinogen-III decarbo	173	472	9	299	204	NF00935425	Methanosar	.0815	24.72	0.56	1.00	.44	5	H	0.62	0.01	0.26
KF	SF001349	uroporphyrinogen-III decarbo	173	472	9	165	269	NF01902752	Rickettsia	.0427	11.78	0.71	1.00	.80	10	H	0.03	0.02	0.41
KF	SF001349	uroporphyrinogen-III decarbo	173	472	9	159	269	NF01902752	Rickettsia	.0427	11.78	0.76	1.00	.86	1	C	0.01	0.01	0.61
QF	SF001357	deoxyribose-phosphate aldola	132	329	6	314	222	232	cellularor	.0735	5.59	0.74	0.63	1.16	12	H	0.06	0.04	0.46
QF	SF001361	3-deoxy-7-phosphoheptulonate	155	399	4	50	289	292	Gammaprote	.0354	15.71	0.32	0.69	2.35	9	H	0.09	0.08	0.19
QF	SF001380	carbamoyl-phosphate synthase	170	479	6	421	291	292	Methanococ	.1187	46.18	0.78	0.77	.49	13	B	0.04	0.11	0.82
FE	SF001391	dTDP-dihydrostreptose syntha	180	372	1	37	315	NF00646303	Pyrococcus	.0972	19.49	0.59	1.00	1.98	13	T	0.04	0.11	0.25
KF	SF001402	glutamate racemase	134	311	1	63	235	236	Bacillales	.0504	7.56	0.92	0.34	1.68	29	T	0.13	0.10	0.24
FK	SF001402	glutamate racemase	134	311	1	278	167	168	Bacteria	.0613	3.88	0.98	0.72	.39	1	B	0.67	0.02	0.19
FQ	SF001430	tRNA-pseudouridine synthase	176	352	1	345	219	220	Cyanobacte	.1178	10.95	0.50	0.97	1.45	10	T	0.04	0.05	0.24
KF	SF001433	threonine synthase	101	583	1	488	159	160	Rhizobiace	.1021	33.74	0.97	0.99	1.22	10	H	0.23	0.04	0.09
FK	SF001433	threonine synthase	101	583	1	352	146	147	Streptococ	.0969	22.87	0.54	0.89	1.24	19	C	0.02	0.05	0.09
EF	SF001440	argininosuccinate lyase	151	489	11	138	291	292	Saccharomy	.0524	18.63	0.33	0.99	2.29	24	H	0.04	0.03	0.31
EF	SF001440	argininosuccinate lyase	151	489	11	39	157	158	Streptococ	.0567	16.60	0.84	0.97	2.02	9	H	0.21	0.03	0.29
EF	SF001455	3-dehydroquinate synthase	132	431	1	219	220	221	Agrobacter	.1105	29.44	0.85	1.00	1.98	19	H	0.20	0.02	0.19
KF	SF001479	xylose isomerase	76	466	27	180	121	127	Gammaprote	.0719	21.17	0.95	0.97	1.35	22	H	0.53	0.07	0.30
KF	SF001484	N-(5'-phospho-D-ribosylformi	109	266	1	10	151	155	Bacteria	.0362	.70	0.43	0.61	1.21	2	B	0.40	0.02	0.33
FQ	SF001493	phosphoglucomutase	129	642	2	199	193	194	Rhizobiace	.0203	10.00	0.35	0.42	2.06	30	B	0.04	0.11	0.09
FQ	SF001493	phosphoglucomutase	129	642	2	439	221	223	Vibrio	.0352	20.73	0.45	0.92	.41	1	B	0.09	0.03	0.03
FE	SF001505	muconate cycloisomerase	97	424	13	86	173	177	Archaea	.0874	7.23	0.38	0.64	1.22	11	T	0.04	0.02	0.19
KF	SF001517	tyrosine-tRNA ligase	178	521	8	184	181	241	Bacteria	.0753	8.00	0.65	0.87	.70	21	T	0.43	0.08	0.04
FE	SF001523	leucine-tRNA ligase	161	1255	1	23	245	246	Ascomycota	.0772	17.31	0.71	0.64	.43	6	T	0.71	0.01	0.18
KF	SF001523	leucine-tRNA ligase	161	1255	1	553	258	277	Bacteria	.1126	70.74	0.97	1.00	1.66	20	H	0.22	0.26	0.28
KF	SF001523	leucine-tRNA ligase	161	1255	1	553	268	269	Rhizobiale	.0373	20.74	0.96	0.99	1.66	20	H	0.22	0.26	0.28
FQ	SF001523	leucine-tRNA ligase	161	1255	1	456	304	305	Xylellafas	.1061	70.77	0.99	1.00	.94	11	H	0.42	0.01	0.33
FQ	SF001528	methionyl-tRNA synthetase, c	115	1011	5	48	215	NF01048563	Streptococ	.1095	52.59	0.99	1.00	2.25	11	B	0.18	0.01	0.39
QF	SF001530	glycine-tRNA ligase alpha ch	95	326	1	220	97	98	Bacteria	.0684	17.91	0.99	0.62	2.25	6	H	0.48	0.13	0.29
QF	SF001530	glycine-tRNA ligase alpha ch	95	326	1	239	96	158	Gammaprote	.0075	1.03	0.28	0.52	3.99	30	H	0.20	0.06	0.06
KF	SF001536	cysteine-tRNA ligase	168	676	1	252	221	222	Spirochaet	.0988	8.66	0.98	1.00	.34	19	B	0.98	0.02	0.00
FK	SF001537	glutamate-tRNA ligase	179	713	2	469	197	198	Alphaprote	.0629	5.46	0.20	0.38	1.68	3	H	0.12	0.04	0.25
FQ	SF001537	glutamate-tRNA ligase	179	713	2	234	218	230	cellularor	.1071	30.53	0.97	0.68	1.36	21	C	0.26	0.08	0.26
FE	SF001547	phenylalanine-tRNA ligase be	143	1034	1	595	224	225	Bacteria	.0759	27.50	0.98	0.28	1.00	11	C	0.41	0.05	0.19
FE	SF001547	phenylalanine-tRNA ligase be	143	1034	1	595	255	256	Cyanobacte	.0489	13.70	0.86	0.38	1.00	11	C	0.41	0.05	0.19
KF	SF001550	acetate-CoA ligase	72	802	1	369	88	89	cellularor	.0429	7.59	0.19	0.28	2.68	0	H	0.04	0.06	0.45
QF	SF001550	acetate-CoA ligase	72	802	1	746	116	132	Fungi/Meta	.0466	14.52	0.33	0.27	2.56	12	B	0.16	0.09	0.33
EF	SF001552	4-coumarate-CoA ligase	173	758	2	747	239	240	cellularor	.0172	4.92	0.33	0.84	1.14	0	H	0.12	0.07	0.42
FE	SF001552	4-coumarate-CoA ligase	173	758	2	162	198	200	Enterobact	.0841	44.84	0.55	0.57	1.34	0	H	0.02	0.08	0.34
FK	SF001552	4-coumarate-CoA ligase	173	758	2	213	217	NF00428215	Lampyrisno	.0894	35.59	0.45	1.00	1.82	25	H	0.01	0.04	0.12
KF	SF001553	succinyl-CoA synthetase, alp	151	467	4	267	204	NF00149549	Susscrofa	.0962	24.60	1.00	1.00	.64	8	B	0.82	0.01	0.12
QF	SF001556	glutamate-ammonia ligase	171	546	2	312	316	NF00881062	Brucellame	.0631	24.44	1.00	1.00	1.47	3	B	0.21	0.19	0.31
FQ	SF001556	glutamate-ammonia ligase	171	546	2	504	315	316	Rhizobiale	.1184	43.34	0.64	1.00	1.15	6	H	0.32	0.04	0.15
FQ	SF001572	phosphoribosylformylglycinam	173	376	1	87	306	313	cellularor	.0490	5.77	0.92	0.53	2.41	28	H	0.17	0.16	0.07
FQ	SF001572	phosphoribosylformylglycinam	173	376	1	87	245	246	Cyanobacte	.0634	7.35	0.81	0.97	2.41	28	H	0.17	0.16	0.07
FK	SF001589	asparagine synthase (glutami	164	827	2	311	184	185	Bacteria	.0226	7.18	0.36	0.23	2.16	24	T	0.23	0.07	0.16
EF	SF001590	carbamoyl-phosphate synthase	166	1446	3	914	270	271	Bacillacea	.0924	55.89	1.00	0.54	.85	28	B	0.64	0.01	0.03
EF	SF001590	carbamoyl-phosphate synthase	166	1446	3	685	270	271	Bacillacea	.0924	55.89	1.00	0.54	.87	17	T	0.62	0.04	0.12
EF	SF001590	carbamoyl-phosphate synthase	166	1446	3	1418	267	279	Bacteria	.0868	79.70	0.21	0.99	3.60	18	T	0.06	0.20	0.09
QF	SF001590	carbamoyl-phosphate synthase	166	1446	3	477	175	180	cellularor	.0418	24.60	0.57	0.57	.75	5	H	0.08	0.57	0.22
FE	SF001590	carbamoyl-phosphate synthase	166	1446	3	592	180	185	cellularor	.0721	68.27	0.95	0.91	2.31	6	H	0.10	0.10	0.43
FE	SF001590	carbamoyl-phosphate synthase	166	1446	3	1407	180	185	cellularor	.0721	68.27	1.00	0.61	2.95	22	H	0.12	0.17	0.09
FQ	SF001590	carbamoyl-phosphate synthase	166	1446	3	477	181	184	Enterobact	.0320	13.17	0.57	0.96	.75	5	H	0.57	0.08	0.22
QF	SF001590	carbamoyl-phosphate synthase	166	1446	3	477	176	NF01076463	Shewanella	.0929	86.78	0.57	1.00	.75	5	H	0.08	0.57	0.22
QF	SF001590	carbamoyl-phosphate synthase	166	1446	3	477	173	NF01057378	Xenorhabdu	.0493	42.88	1.00	1.00	.75	5	H	0.08	0.57	0.22
QF	SF001591	human acetyl-CoA carboxylase	22	880	2	470	38	39	Saccharomy	.0567	20.60	0.33	0.87	1.99	13	B	0.09	0.09	0.05
FK	SF001619	biotin synthetase	127	440	1	278	190	192	Bacillus	.1094	29.31	0.93	0.98	1.84	20	H	0.14	0.05	0.32
QF	SF001734	transcriptional regulator, E	27	276	3	180	34	36	Eukaryota	.0780	10.37	0.97	1.00	1.16	27	C	0.41	0.26	0.23
KF	SF002173	rat ribosomal protein L36a	63	105	1	88	86	87	Eukaryota	.0742	6.36	0.88	0.98	1.70	28	B	0.37	0.05	0.08
FK	SF002212	rat ribosomal protein L31	44	100	2	34	77	78	Euryarchae	.0820	1.56	0.18	0.15	.92	23	H	0.07	0.02	0.16
QF	SF002327	tropomyosin	70	284	1	263	126	127	Eumetazoa	.0235	2.02	1.00	0.47	1.95	12	H	0.32	0.01	0.00
FQ	SF002328	myosin heavy chain	32	1406	4	1354	39	44	Muridae	.0898	43.59	0.73	1.00	.85	16	H	0.34	0.09	0.47
FQ	SF002347	villin	49	951	1	745	94	NF00963318	Arabidopsi	.0379	26.75	1.00	1.00	.23	3	B	0.08	0.02	0.00
FE	SF002347	villin	49	951	1	747	96	NF00679426	Arabidopsi	.0121	10.00	1.00	1.00	1.61	24	C	0.15	0.02	0.08
FK	SF002347	villin	49	951	1	6	71	72	Euteleosto	.0533	26.82	0.73	0.97	.53	10	H	0.62	0.04	0.23
FQ	SF002390	lipid binding protein, FABP-	166	149	24	7	216	217	Eutheria	.1188	12.11	0.97	1.00	.28	9	T	0.81	0.03	0.09
EF	SF002401	GTP-binding protein obg	55	440	1	415	83	84	Bacilli	.1007	16.91	0.28	0.52	2.94	29	H	0.11	0.15	0.15
KF	SF002520	serum albumin	31	600	5	374	44	NF00395706	Xenopuslae	.0806	42.72	0.94	1.00	1.45	12	T	0.10	0.03	0.06
FQ	SF002533	thyroid hormone receptor	22	268	8	266	25	NF00379912	Avianeryth	.0685	17.62	1.00	1.00	1.84	30	T	0.73	0.09	0.05
QF	SF002533	thyroid hormone receptor	22	268	8	259	40	NF00560340	Rattusnorv	.0242	5.23	0.88	1.00	1.35	24	C	0.09	0.05	0.00
FQ	SF002533	thyroid hormone receptor	22	268	8	262	42	NF00558613	Rattusnorv	.0228	6.00	1.00	1.00	1.93	10	H	0.73	0.09	0.00
QF	SF002549	transferrin	21	785	11	358	28	32	Euteleosto	.0544	7.42	0.36	0.31	3.64	6	H	0.14	0.10	0.10
EF	SF002583	heat shock protein 90	75	358	12	77	117	118	Eukaryota	.0579	9.31	0.50	0.70	2.41	24	H	0.15	0.13	0.15
EF	SF002585	heat shock protein dnaJ	140	312	2	187	170	173	Alphaprote	.0807	12.22	0.41	0.97	1.38	22	B	0.10	0.06	0.38
FK	SF002699	rRNA N-glycosidase	103	348	19	132	141	NF00739837	Geloniummu	.0603	13.00	1.00	1.00	.41	14	T	0.62	0.01	0.20
FE	SF002740	lysine-arginine-ornithine-bi	135	308	4	56	154	NF01995554	Burkholder	.0217	5.00	1.00	1.00	.28	0	H	0.70	0.01	0.22
EF	SF002741	dipeptide transport protein	171	762	4	752	201	209	cellularor	.0616	11.22	0.36	0.68	1.33	7	C	0.04	0.14	0.41
QF	SF002741	dipeptide transport protein	171	762	4	208	201	209	cellularor	.0616	11.22	0.24	0.49	1.56	19	C	0.02	0.11	0.11
QF	SF002821	long-chain fatty acid transp	82	522	1	218	102	NF00735036	Haemophilu	.0465	24.04	0.84	1.00	3.25	2	T	0.08	0.12	0.04

FQ	SF002854	lipoprotein-28	182	347	1	127	219	260	Bacteria	.0994	20.01	0.93	0.64	1.71	30	H	0.30	0.23	0.11
EF	SF002854	lipoprotein-28	182	347	1	148	219	260	Bacteria	.0994	20.01	0.96	0.91	1.92	0	C	0.33	0.10	0.16
FQ	SF002854	lipoprotein-28	182	347	1	127	225	226	Bacteria	.1151	11.01	1.00	0.75	1.71	30	H	0.30	0.23	0.11
FE	SF002854	lipoprotein-28	182	347	1	318	268	269	Proteobact	.0257	3.25	0.64	0.44	1.02	12	H	0.43	0.04	0.21
KF	SF002945	Parasporal crystal protein	107	786	3	514	128	129	Bacillusth	.0578	15.10	0.81	0.94	1.59	25	B	0.07	0.06	0.24
QF	SF002945	Parasporal crystal protein	107	786	3	70	143	144	Bacillusth	.0199	2.24	0.56	0.32	.93	13	H	0.23	0.51	0.03
FQ	SF003012	translation initiation facto	61	662	1	272	62	NF00723453	Vibriochol	.0885	35.99	0.99	1.00	3.21	13	B	0.11	0.03	0.00
KF	SF003068	isoamylase-type debranching	108	926	1	210	163	164	Sulfolobus	.0394	8.54	0.33	0.59	.54	17	C	0.01	0.06	0.34
FE	SF003103	murG protein	135	468	1	322	227	228	Bacilli	.0535	8.44	0.45	0.43	2.44	21	T	0.07	0.09	0.29
FE	SF003103	murG protein	135	468	1	169	136	221	Bacteria	.0519	3.66	0.86	0.50	.87	19	T	0.44	0.14	0.17
KF	SF003103	murG protein	135	468	1	173	162	179	Proteobact	.0713	10.15	0.67	0.46	1.78	24	T	0.20	0.23	0.18
FQ	SF003315	molybdenum cofactor precurso	106	200	2	33	140	141	Cyanobacte	.1154	11.27	0.97	0.30	2.42	11	B	0.25	0.03	0.09
EF	SF003315	molybdenum cofactor precurso	106	200	2	184	154	155	Proteobact	.0824	6.01	1.00	0.84	1.47	9	B	0.46	0.03	0.06
KF	SF003376	polyomavirus coat protein VP	112	388	4	158	206	207	Polyomavir	.0530	5.26	0.50	0.86	1.55	25	C	0.83	0.06	0.01
KF	SF004093	cucumber mosaic virus coat p	136	219	2	194	250	NF01219168	Cucumbermo	.0039	1.00	1.00	1.00	2.30	18	C	0.18	0.01	0.01
KF	SF004274	phage T4 DNA polymerase acce	92	480	3	468	112	113	Bilateria	.1129	18.79	0.23	0.85	2.42	23	H	0.05	0.03	0.25
QF	SF004274	phage T4 DNA polymerase acce	92	480	3	435	133	134	Saccharomy	.0824	16.34	0.48	0.99	1.34	24	H	0.14	0.03	0.30
EF	SF004717	uncharacterized conserved pr	31	225	1	161	36	37	cellularor	.0401	2.93	0.44	0.22	1.56	25	H	0.23	0.13	0.26
FK	SF004741	hypothetical protein ywpJ	46	322	2	158	53	54	Firmicutes	.0988	5.39	0.23	0.22	1.78	28	H	0.20	0.09	0.15
FQ	SF005293	peptide methionine sulfoxide	49	273	1	258	60	NF00300731	Schizosacc	.0586	6.82	0.73	1.00	1.13	22	C	0.02	0.04	0.06
KF	SF005418	conserved hypothetical prote	64	125	2	30	68	69	Bacillales	.0753	4.93	0.33	0.67	.93	1	B	0.16	0.06	0.09
FQ	SF005529	NADH peroxidase	99	510	6	154	101	102	Spirochaet	.1138	27.42	0.96	0.52	1.46	17	C	0.16	0.01	0.25
FE	SF005531	Peptidase V	122	534	1	411	133	134	Bacillacea	.0224	8.48	0.64	0.59	1.73	8	H	0.12	0.03	0.37
QF	SF005572	nitrogen fixation protein ni	87	469	1	173	115	117	Bacteria	.0663	10.14	0.99	0.98	.97	8	C	0.69	0.10	0.06
KF	SF005591	auxin-induced protein	177	309	1	197	234	239	eurosidsI	.1098	12.91	0.91	0.78	1.52	28	H	0.20	0.02	0.05
KF	SF005604	endoxyloglucan transferase	96	321	1	320	113	116	Magnolioph	.0240	4.68	0.44	0.67	1.00	1	C	0.05	0.12	0.05
FK	SF005639	conserved hypothetical prote	27	254	1	102	40	43	cellularor	.0539	3.12	0.43	0.30	1.56	1	H	0.37	0.07	0.07
FE	SF005677	pyrophosphate-dependent phos	40	602	1	371	47	50	cellularor	.1073	26.95	1.00	0.33	2.10	2	H	0.23	0.30	0.21
KF	SF005677	pyrophosphate-dependent phos	40	602	1	492	41	46	Spirochaet	.0194	8.08	0.57	0.82	1.12	16	C	0.42	0.05	0.38
EF	SF005783	conserved hypothetical prote	74	281	1	246	89	90	cellularor	.0648	5.37	0.61	0.74	1.85	13	B	0.18	0.15	0.18
FE	SF005837	nifS protein	168	549	4	141	184	NF00482406	Mycobacter	.0938	36.75	1.00	1.00	.47	0	H	0.03	0.03	0.02
EF	SF005855	lipopolysaccharide biosynthe	148	420	3	96	207	208	Bacteria	.0145	3.24	0.96	0.99	1.30	15	T	0.46	0.18	0.17
FE	SF005855	lipopolysaccharide biosynthe	148	420	3	282	272	273	Bacteria	.0423	15.66	0.95	0.71	1.21	17	H	0.09	0.04	0.40
FK	SF005855	lipopolysaccharide biosynthe	148	420	3	283	272	278	Bacteria	.0443	7.27	0.38	0.46	.68	4	H	0.14	0.09	0.62
FK	SF005855	lipopolysaccharide biosynthe	148	420	3	263	278	279	Bacteria	.0785	15.57	0.55	0.98	1.01	7	T	0.09	0.03	0.35
KF	SF005855	lipopolysaccharide biosynthe	148	420	3	179	222	223	Bacteroide	.0481	10.54	0.55	0.79	2.04	20	H	0.11	0.01	0.22
KF	SF005909	acriflavin resistance protei	50	1141	2	843	51	52	Bacteria	.0487	28.52	0.26	0.72	1.21	28	H	0.06	0.38	0.26
KF	SF006008	methicillin resistance facto	58	436	1	369	62	107	Streptococ	.0567	6.59	0.74	0.95	.78	23	B	0.09	0.10	0.00
KF	SF006038	Succinyl-diaminopimelate des	136	526	1	120	221	229	Gammaprote	.0386	6.87	0.55	0.65	1.85	13	T	0.04	0.09	0.20
KF	SF006052	methionyl-tRNA formyltransfe	147	411	1	372	199	200	Mycoplasma	.0619	8.85	0.83	0.97	.49	3	T	0.01	0.01	0.07
FQ	SF006089	fimbrial protein fimH	34	293	2	95	58	NF00695230	Escherichi	.1013	18.13	0.42	1.00	1.72	24	C	0.09	0.03	0.09
FE	SF006150	Hypothetical protein HI0333	149	655	1	150	153	154	Bacillus	.0596	15.75	0.95	0.78	1.48	23	H	0.26	0.01	0.21
FK	SF006150	Hypothetical protein HI0333	149	655	1	639	267	NF00366299	Saccharomy	.0673	31.00	1.00	1.00	.16	26	T	0.96	0.01	0.03
QF	SF006241	conserved hypothetical prote	112	339	2	198	131	132	cellularor	.0339	3.54	0.39	0.33	1.34	0	H	0.17	0.06	0.28
KF	SF006241	conserved hypothetical prote	112	339	2	218	113	203	Proteobact	.0716	12.33	1.00	0.93	1.13	25	B	0.23	0.22	0.02
KF	SF006241	conserved hypothetical prote	112	339	2	218	115	118	Proteobact	.0281	6.30	0.82	0.87	1.13	25	B	0.23	0.22	0.02
FQ	SF006294	phosphoenolpyruvate carboxyk	90	600	5	135	118	119	cellularor	.0322	6.53	0.36	0.93	2.12	6	B	0.18	0.19	0.01
EF	SF006305	septum formation protein maf	145	234	1	158	260	261	Bacilli	.0717	5.01	0.46	0.31	2.25	8	B	0.17	0.09	0.17
KF	SF006305	septum formation protein maf	145	234	1	118	254	258	cellularor	.0294	3.62	0.32	0.29	1.85	6	H	0.09	0.05	0.28
FK	SF006305	septum formation protein maf	145	234	1	225	215	216	Rhizobiale	.0807	4.71	0.39	0.97	.72	2	H	0.03	0.03	0.72
FK	SF006353	DNA-directed DNA polymerase	44	357	1	304	77	78	Bacilli	.0396	1.35	0.41	0.30	1.30	10	T	0.21	0.10	0.33
FQ	SF006356	arginine deiminase arcA	67	436	1	93	74	75	Bacteria	.0423	8.34	0.81	0.33	1.11	0	H	0.39	0.08	0.19
FQ	SF006356	arginine deiminase arcA	67	436	1	93	130	NF01168110	Streptococ	.0386	15.94	1.00	1.00	1.11	0	H	0.39	0.08	0.19
FQ	SF006496	Salmonella typhimurium ribor	63	776	1	86	114	115	Mycoplasma	.0428	23.99	1.00	1.00	.94	14	H	0.62	0.08	0.07
FK	SF006615	Zn-dependent carboxypeptidas	52	539	1	465	74	75	Bacillus	.0441	10.33	0.88	0.98	1.59	17	H	0.25	0.21	0.42
EF	SF006615	Zn-dependent carboxypeptidas	52	539	1	156	53	71	cellularor	.0817	10.65	0.31	0.18	1.57	9	H	0.27	0.29	0.12
KF	SF006650	Bacillus subtilis ATP phosph	50	249	1	97	93	96	Bacteria	.0282	3.80	0.82	0.56	2.11	18	B	0.30	0.04	0.16
EF	SF006765	4-diphosphocytidyl-2-methyl-	116	295	3	269	193	194	Bacteria	.1004	2.22	0.24	0.42	1.90	18	T	0.08	0.06	0.41
FK	SF006765	4-diphosphocytidyl-2-methyl-	116	295	3	84	224	228	Cyanobacte	.0622	4.72	0.50	0.48	1.68	11	T	0.27	0.05	0.23
EF	SF006766	Haemophilus influenzae conse	30	174	1	35	32	33	Gammaprote	.0632	4.37	0.54	0.99	1.62	23	H	0.30	0.20	0.13
EF	SF006766	Haemophilus influenzae conse	30	174	1	79	41	42	Rhizobiale	.1178	2.26	0.51	0.98	.74	22	C	0.57	0.07	0.14
FQ	SF009283	4-hydroxyphenylpyruvate_diox	74	546	2	227	141	NF00149975	Susscrofa	.1141	39.00	1.00	1.00	.28	28	C	0.73	0.01	0.21
KF	SF009316	Saccharomyces hypothetical p	23	400	1	53	41	NF00682507	Arabidopsi	.0116	4.97	1.00	1.00	1.16	6	H	0.09	0.04	0.04
KF	SF009393	ornithine/lysine/arginine de	67	914	2	790	97	98	Lactobacil	.0321	9.91	0.81	0.73	1.46	16	H	0.31	0.09	0.01
FE	SF009407	isocitrate dehydrogenase, mo	36	743	1	619	51	52	Actinobact	.0449	20.24	0.47	1.00	.98	2	H	0.03	0.06	0.78
FK	SF015568	human signal transducer and	74	1001	2	845	138	139	Eutheria	.0129	9.91	1.00	1.00	1.03	10	C	0.16	0.07	0.08
FQ	SF015601	methyltransferase, slr0722 t	110	343	2	49	166	167	Rhizobiace	.0733	20.41	0.87	0.80	1.08	18	B	0.11	0.02	0.21
FE	SF015689	acetaldehyde dehydrogenase (	44	318	1	69	52	53	Bacteria	.0995	14.80	0.95	0.55	1.60	14	H	0.43	0.17	0.23
FK	SF015858	Not Yet Assigned	65	266	1	200	100	101	Cyanobacte	.1139	8.87	0.28	0.54	1.00	19	B	0.72	0.06	0.17
EF	SF016033	esterase	92	315	2	302	167	NF00674079	Arabidopsi	.0725	11.61	0.96	1.00	1.19	26	H	0.07	0.07	0.37
FK	SF016187	Pyrococcus abyssi hypothetic	37	529	1	323	46	48	Proteobact	.0403	7.95	0.30	0.34	3.00	20	H	0.05	0.11	0.21
EF	SF016203	Pyrococcus horikoshii hypoth	18	591	1	179	23	24	Actinobact	.0600	19.69	0.70	1.00	.62	0	H	0.06	0.11	0.00
FE	SF016516	allantoicase	15	406	1	36	23	NF01976658	Burkholder	.0914	18.42	0.98	1.00	1.51	7	B	0.36	0.14	0.00
QF	SF016528	Not Yet Assigned	31	512	1	488	53	54	Gammaprote	.0474	12.93	0.45	0.95	2.19	26	H	0.10	0.10	0.58
QF	SF016705	Not Yet Assigned	63	331	1	264	75	80	Bacteria	.0746	5.21	0.26	0.30	1.85	17	H	0.10	0.17	0.21
FE	SF016882	Not Yet Assigned	33	499	1	307	37	52	Bacteria	.0448	5.59	0.56	0.58	1.50	17	H	0.30	0.24	0.06
EF	SF019142	alkanesulfonate monooxygenas	21	408	1	375	26	27	Bacillus	.0571	14.07	0.29	0.96	2.75	22	B	0.24	0.10	0.39
FQ	SF019659	2'-hydroxyisoflavone reducta	51	370	2	306	67	NF00538070	Thujaplica	.1171	29.58	1.00	1.00	1.08	28	H	0.18	0.02	0.33
KF	SF027476	magnesium chelatase, subunit	56	384	1	292	93	94	cellularor	.0340	2.18	0.24	0.30	1.81	13	H	0.50	0.04	0.14
FQ	SF036351	Alcaligenes eutrophus nitrat	56	1055	1	716	92	NF01831474	Photobacte	.0778	56.83	1.00	1.00	.81	29	T	0.49	0.02	0.39
FE	SF036421	tricorn protease	18	1271	2	1112	28	29	Actinobact	.0830	10.82	0.72	0.37	.90	25	T	0.44	0.11	0.11
FQ	SF036492	aldehyde dehydrogenase	121	639	1	34	184	185	Bacteria	.0967	26.14	0.50	0.96	1.21	27	H	0.37	0.09	0.08
FQ	SF036492	aldehyde dehydrogenase	121	639	1	293	218	219	Firmicutes	.0300	5.00	0.99	0.78	.72	2	H	0.55	0.07	0.09
FK	SF036492	aldehyde dehydrogenase	121	639	1	293	227	235	Gammaprote	.0783	12.74	0.89	0.35	.72	2	H	0.55	0.01	0.21
FE	SF036497	homoserine dehydrogenase lac	46	390	2	257	66	67	Pyrococcus	.0318	2.99	0.64	0.99	1.17	13	T	0.09	0.13	0.43
FQ	SF036501	copper chaperone for superox	23	309	4	199	25	27	Eukaryota	.0718	6.86	1.00	0.36	1.41	30	B	0.26	0.04	0.04
FE	SF036520	leghemoglobin	91	173	3	123	124	NF00718154	Glycinemax	.0328	3.55	1.00	1.00	.28	20	H	0.99	0.01	0.00
QF	SF036546	pyruvate synthase, PorD subu	95	145	24	32	153	154	Euryarchae	.0622	1.32	0.35	0.38	.45	4	C	0.03	0.05	0.16
EF	SF036578	DNA replication factor C, la	26	601	1	279	38	NF00557796	Rattusnorv	.0701	17.18	0.39	1.00	1.21	25	H	0.04	0.04	0.12
KF	SF036602	DNA polymerase IV	120	514	3	40	167	169	Mycoplasma	.0646	15.26	0.85	0.79	.83	0	B	0.55	0.02	0.27
QF	SF036602	DNA polymerase IV	120	514	2	389	167	169	Mycoplasma	.0646	15.26	0.15	0.16	2.34	29	H	0.03	0.05	0.23
FQ	SF036653	fumarate reductase, flavopro	77	773	15	491	135	136	Bacilli	.0416	11.48	0.35	0.95	1.76	2	B	0.16	0.10	0.06
FE	SF036771	alpha-amylase/trypsin inhibi	37	160	4	107	39	40	Poaceae	.0668	8.90	1.00	0.98	1.98	18	B	0.12	0.34	0.18
KF	SF036776	unsaturated glucuronyl hydro	18	407	1	340	19	20	Bacteria	.0990	13.16	0.94	0.53	1.36	15	H	0.11	0.17	0.33
FQ	SF036798	GTP-binding protein era homo	101	364	1	196	130	131	Bacteria	.0897	7.90	1.00	0.43	.59	12	C	0.62	0.05	0.04
FE	SF036798	GTP-binding protein era homo	101	364	1	235	185	186	Mycoplasma	.0837	2.34	0.66	0.53	.38	26	C	0.03	0.05	0.09
QF	SF500069	benzoylformate decarboxylase	27	585	1	438	39	40	Proteobact	.0517	8.02	0.31	0.36	1.12	8	C	0.12	0.27	0.16
FK	SF500076	L-aspartate oxidase (quinoli	89	613	1	211	93	NF00658828	Arabidopsi	.0294	19.01	1.00	1.00	1.22	7	B	0.19	0.01	0.57
FE	SF500077	adenylylsulfate reductase, a	11	682	1	527	18	NF00579937	Pseudomona	.0784	33.67	0.64	1.00	1.23	0	H	0.09	0.09	0.54
QF	SF500079	uroporphyrinogen-III decarbo	134	451	9	164	243	NF00172073	Chlamydiam	.0966	23.62	1.00	1.00	.40	18	T	0.02	0.01	0.75

FQ	SF500079	uroporphyrinogen-III decarbo	134	451	9	224	177	181		Proteobact	.1191	14.78	0.82	0.88	.87	1	C	0.37	0.07	0.45
FK	SF500116	azurin	30	133	26	16	49	NF01327006		Burkholder	.0209	3.00	1.00	1.00	.50	14	C	0.77	0.03	0.20
FK	SF500151	cytochrome c2	50	151	10	91	93	94		Proteobact	.0888	3.42	0.40	0.86	1.50	25	T	0.32	0.22	0.10
FE	SF500163	zinc metalloendopeptidase, r	63	395	5	46	69	79		Bacteria	.0880	13.60	0.84	0.54	2.33	24	B	0.25	0.14	0.16
FQ	SF500169	aspartate carbamoyltransfera	169	416	11	187	284	286		Bacteria	.0246	4.16	0.95	0.88	1.23	25	T	0.34	0.14	0.17
FK	SF500169	aspartate carbamoyltransfera	169	416	11	187	220	221		Proteobact	.0490	4.80	0.99	0.66	1.23	25	T	0.34	0.20	0.21
FK	SF500169	aspartate carbamoyltransfera	169	416	11	187	194	195		Rhizobiale	.0585	8.86	0.57	1.00	1.23	25	T	0.34	0.20	0.21
