# Supplementary material for: Integrating protein structures and precomputed genealogies in the Magnum database: Examples with cellular retinoid binding proteins
Source: BMC Bioinformatics. 2006 Feb 23;7:89. doi: 10.1186/1471-2105-7-89 (PMC1475641; doi:10.1186/1471-2105-7-89)
Supplement: Additional File 3 — Multiple sequence alignment of cellular retinoid binding proteins. Aligned sequences ordered according to the tree shown in Figure 6. The sequence of the reference structure PDB:1opbA has been underlined. [file 1471-2105-7-89-S3.pdf]

Dataset S3. Multiple sequence alignment of cellular retinoic acid binding proteins and cellular retinol binding proteins.

```
              00000000000000000000000000000000000000000000000000
align sites   00000000011111111112222222222333333333344444444445
              12345678901234567890123456789012345678901234567890

              ...000000000000000000000000..000000000000000000000
PDB:1opbA     ...000000000111111111122222..222223333333333444444
              ...123456789012345678901234..567890123456789012345

retinoic acid subfam 1
NF00163989    ---MPNFAGTWKMRSSENFDELLKALGVNAMLRKVAVAAASKPHVEIRQD
NF00101210    ---MPNFAGTWKMRSSENFDELLKALGVNAMLRKVAVAAASKPHVEIRQD
NF00048039    --------------GSENFDELLKALGVNAMLRKVAVAAASKPHVEIRQD
NF00048311    ---MPNFARTWKMRSSENFDELLKALGVNAMLRKVAVAAASKPHVEIRQD
NF01354485    --MPPNFAGTWKMKSSENFDELLKALGVNAMLRKVAGAAAAKPHVEIRQD
NF00393248    ---MPNFAGTWKMKSSENFDELLKALGVNTMLRKVAVAAASNPHVEIRQD

retinoic acid subfam 2
NF00517003    ---MPNFSGNWKIIRSENFEEMLKALGVNMMMRKIAVAAASKPAVEIKQE
NF00521976    ---MPNFSGNWKIIRSENFEEMLKALGVNMMMRKIAVAAASKPAVEIKQE
NF00560143    ---MPNFSGNWKIIRSENFEEMLKALGVNMMMRKIAVAAASKPAVEIKQE
NF00099520    ---MPNFSGNWKIIRSENFEELLKVLGVNVMLRKIAVAAASKPAVEIKQE
NF00397245    ---MPNFSGHWKMKQSENFEEMLKALGVNLMLRKIAVAAASKPAVEIKQE
NF00397835    ---MPNFSGHWKMKQSENFEEMLKALGVNLMLRKIAVAAASKPAVEIKQE
NF01250802    DRKIPDFAGTWKMKSSENFEELLKALGVNVMLRKIAVAAASKPSVEITQE
NF00047806    ---MPNFAGTWKMRSSENFDELLKALGVNAMLRKVAVAAASKPHVEIRQD

retinol binding protein subfam 2 10 nM
              --cccccceeeeeeeeechhhhhhhcccchhhhhhhccccc--eeeeeee
NF00530124    --MTKDQNGTWEMESNENFEGYMKALDIDFATRKIAVRLTQ--TKIITQD
NF00559288    --MTKDQNGTWEMESNENFEGYMKALDIDFATRKIAVRLTQ--TKIIVQD
NF00132596    --MTRDQNGTWEMESNENFEGYMKALDIDFATPKIAVRLTQ--TKVIDQD
NF00150317    --MTRDQNGTWEMESNDNFEGYMKALDIDFATRKIAVALTQ--TKIIEQD
NF00826980    --MPADFNGTWEMLSNDNFEDVMKALDIDFATRKIAVHLKQ--TKVIVQN

retinol binding protein subfam 4 200 nM
NF00510017    --MPADLSGTWNLLSSDNFEGYMLALGIDFATRKIAKLLKP--QKVIEQN
NF00137740    --MPADLSGTWTLLSSDNFEGYMLALGIDFATRKIAKLLKP--QKVIEQN

retinol binding protein subfam 1 0.1 nM
NF00567514    --MPVDFNGYWKMLSNENFEEYLRALDVNVALRKIANLLKP--DKEIVQD
NF00517150    --MPVDFNGYWKMLSNENFEEYLRALDVNVALRKIANLLKP--DKEIVQD
NF00160809    ---PVDFTGYWKMLANENFEEYLRALDVNVALRKIANLLKP--DKEIVQE
NF00078388    --MPVDFTGYWKMLVNENFEEYLRALDVNVALRKIANLLKP--DKEIVQD

retinol binding protein subfam 3 60 nM
NF00043544    ---------YYRFVSQDNMDNYLRVLDINVALRKLVCLLKP--DKEIIHN
NF00113102    --MPPNLTGYYRFVSQKNMEDYLQALNISLAVRKIALLLKP--DKEIEHQ
              xxxxxxxxxxxxxxxxx*xxxxxxxxxxxxxx**xxxxxxxxxxxx*x|x
```

```
                 00000000000000000000000000000000000000000000000001
align sites      55555555566666666667777777777888888888899999999990
                 12345678901234567890123456789012345678901234567890

                 00.....000000000000000000000000000000000000000000
PDB:1opbA        44.....445555555555666666666677777777778888888889
                 67.....890123456789012345678901234567890123456789 0

retinoic acid subfam 1
NF00163989       GD-----QFYIKTSTTVRTTEINFKVGEGFEEETV--DGRKCRSLPTWEN
NF00101210       GD-----QFYIKTSTTVRTTEINFKVGEGFEEETV--DGRKCRSLATWEN
NF00048039       GD-----QFYIKTSTTVRTTEINFKIGESFEEETV--DGRKCRSLATWEN
NF00048311       GD-----QFYIKTSTTVRTTEINFKIGESFEEETV--DGRKCRSLATWEN
NF01354485       GE-----QFYIKTSTTVRTTEINFRVGEEFDEETV--DGRKCKSLATWET
NF00393248       GE-----KFYIKTSTTVRTTEINFHIGEEFDEETV--DGRKCKSLPTWES

retinoic acid subfam 2
NF00517003       ND-----NFYIKTSTTVRTTEINFKIGEEFEEQTV--DGRPCKSLVKWES
NF00521976       ND-----TFYIKTSTTVRTTEINFKIGEEFEEQTV--DGRPCKSLVKWES
NF00560143       NDD----TFYIKTSTTVRTTEINFKIGEEFEEQTV--DGRPCKSLVKWES
NF00099520       GD-----TFYIKTSTTVRTTEINFKVGEEFEEQTV--DGRPCKSLVKWES
NF00397245       GE-----TFYIKTSTTVRTTEINFKIGEEFDEQTV--DGRPCKSLAKWVS
NF00397835       GE-----TFYIKTSTTVRTTEINFKLGGGFDEQTV--DGRNCRSLPEWEN
NF01250802       GE-----TLTIKTSTSVRTTNVTFTVGQEFNEATV--DGRPCTSFPRWVT
NF00047806       GD-----QFYIKTSTTVRTTEINFKIGESFEEETV--DGRKCRSLATWQN

retinol binding protein subfam 2 10 nM
                 cc-----eeeeeeeccccceeeeeeccceeeecccccccceeeeeeeeec
NF00530124       GD-----NFKTKTNSTFRNYDLDFTVGVEFDEHTKGLDGRHVKTLVTWEG
NF00559288       GD-----NFKTKTNSTFRNYDLDFTVGVEFDEHTKGLDGRNVKTLVTWEG
NF00132596       GD-----NFKTKTTSTFRNYDVDFTVGVEFDEYTKSLDNRHVKALVTWEG
NF00150317       GD-----KFKTKTNSTFRNYDLDFTVGVEFDEYTKGLDNRNVKTLIIWEG
NF00826980       GD-----KFETKTLSTFRNYEVNFVIGEEFDEQTKGLDNRTVKTLVKWDG

retinol binding protein subfam 4 200 nM
NF00510017       GD-----SFTIQTCSSLRNYLVKFKVGEEFEEDNKGLDNRKCTSLVTWEN
NF00137740       GD-----SFTIHTNSSLRNYFVKFKVGEEFDEDNRGLDNRKCKSLVIWDN

retinol binding protein subfam 1 0.1 nM
NF00567514       GD-----HMIIRTLSTFRNYIMDFQVGKEFEEDLTGIDDRKCMTTVSWDG
NF00517150       GD-----HMIIRTLSTFRNYIMDFQVGKEFEEDLTGIDDRKCMTTVSWDG
NF00160809       GD-----HMIIRTLSTFRNYIMDFQVGKEFEEDLTGIDDRKCMTTVSWDG
NF00078388       GD-----HMIIRTLSTFRNYIMDFQVGKEFEEDLTGIDDRKCMTTVSWDG

retinol binding protein subfam 3 60 nM
NF00043544       GN-----HMTIRTLTTLRNYIMDFDLGTEFEEDLGPVDGRKCQTIVQWNG
NF00113102       GN-----HMTVRTLSTFRNYTVQFDVGVEFEEDLRSVDGRKCQTIVTWEE
                 |x-----xxxxxxxxxx*xxxxxxxx*xxxx*xxxxxxxxxxxxxxx*xx
```

```
              1111111111111111111111111111111111111111111111111
align sites   0000000001111111111222222222233333333334444444444
              1234567890123456789012345678901234567890123456789

              00....000000011111111111111111111111111111111111
PDB:1opbA     99....9999999000000000000001111.1111112222222222233333
              12....345678901234567890123.456789012345678901234

retinoic acid subfam 1
NF00163989    ENKIHCTQTLLEGDGPKTYWTRELAND-ELILTFGADDVVCTRIYVRE-
NF00101210    ENKIHCTQTLLEGDGPKTYWTRELAND-ELILTFGADDVVCTRIYVRE-
NF00048039    ENKIYCKQTLIEGDGPKTYWT----------------------------
NF00048311    ENKIYCKQTLIEGDGPKTYWTRELAND-ELIL-----------------
NF01354485    ENKMYCKQTLVDGDGPVTYWSRELRGD-ELILIFGADDVVCTRIYVRE-
NF00393248    ENKIRCKQTLVEGDGPKTFWTRELNGD-ELTLVFGADDVVCTRIYVRE-

retinoic acid subfam 2
NF00517003    GNKMVCEQRLLKGEGPKTSWSRELTNDGELILTMTADDVVCTRVYVRE-
NF00521976    GNKMVCEQRLLKGEGPKTSWSRELTNDGELILTMTADDVVCTRVYVRE-
NF00560143    ENKMVCEQRLLKGEGPKTSWSRELTNDGELILTMTADDVVCTRVYVRE-
NF00099520    ENKMVCEQKLLKGEGPKTSWTRELTNDGELILTMTADDVVCTRVYVRE-
NF00397245    ENKMACEQKLLKGDGPKTAWTREMTNDGELILTMTADDVVCTRIYIRD-
NF00397835    ENKIHCTQTVLEGEGPKTSWTRELANDEALILTMTADDVVCTRIYVREL
NF01250802    DSKISCEQTLQKGEGPKTSWTREITNDAELILTMTADDVVCTRVYVRE-
NF00047806    EN------KIH--------------------------------------

retinol binding protein subfam 2 10 nM
              ce----eeeeeecccccceeeeeeecc-eeeeeeeecceeeeeeeeeec
NF00530124    NT----LVCVQKGEKENRGWKQWVEGD-KLYLELTCGDQVCRQVFKKK-
NF00559288    NT----LVCVQKGEKENRGWKQWVEGD-KLYLELTCGDQVCRQVFKKK-
NF00132596    DV----LVCVQKGEKENRGWKQWIEGD-KLYLELTCGDQVCRQVFKKK-
NF00150317    DA----LVCVQKGEKENRGWKQWVEGD-KLYLELTCGDQVCRQVFKKK-
NF00826980    DK----LVCVQKGEKENRGWKQWIEGD-LLHLEIHCQDKVCHQVFKKKN

retinol binding protein subfam 4 200 nM
NF00510017    DK----LTCVQRGEKKNRGWSHWIEGD-QLHLEMFCEGQVCKQTFQRA-
NF00137740    DR----LTCIQKGEKKNRGWTHWIEGD-KLHLEMFCEGQVCKQTFQRA-

retinol binding protein subfam 1 0.1 nM
NF00567514    DK----LQCVQKGEKEGRGWTQWIEGD-ELHLEMRAEGVTCKQVFKKVH
NF00517150    DK----LQCVQKGEKEGRGWTQWIEGD-ELHLEMRAEGVICKQVFKKVH
NF00160809    DK----LECVQKGEKEGRGWTQWIEGD-ELHLLMRVEGVVCKQVFKKVH
NF00078388    DK----LQCVQKGEKEGRGWTQWIEGD-ELHLEMRVEGVVCKQVFKKVQ

retinol binding protein subfam 3 60 nM
NF00043544    DK----LVCEQHGEKKNRGWKQWLEGD-FLHLHMSAEDETCIQIFEKIK
NF00113102    EH----LVCVQKGEVPNRGWRHWLEGE-MLYLELTARDAVCEQVFRKVR
              xxxxxxxxxxxx*xxxxxxxxxxxxx|xxxxxxxxxxxxxxxxxxxxxx
```
