# Supplementary material for: Integrating protein structures and precomputed genealogies in the Magnum database: Examples with cellular retinoid binding proteins
Source: BMC Bioinformatics. 2006 Feb 23;7:89. doi: 10.1186/1471-2105-7-89 (PMC1475641; doi:10.1186/1471-2105-7-89)
Supplement: Additional File 4 — Amino acid replacement report. Amino acid replacements for the seven labeled branches in Figure 6. [file 1471-2105-7-89-S4.pdf]

Table S5. CRBP amino acid replacements.

Align: site numbers in the alignment.
PDB: positions in the protein chain PDB:1opbA. (e/b = exposed/buried; C/H/T/B = Coil/Helix/Turn/Strand)
Branch: {parent probability} parentchild residues {child probability}; probabilities below 70% shown)

Align	PDB	Branch 1	Branch 2	Branch 3	Branch 4	Branch 5	Branch 6	Branch 7
1	.	.	.	.	.	.	.	.
2	.	.	.	.	.	.	.	.
3	.	.	.	.	.	.	.	.
4	1eC	MP	.	PT	.	.	.	.
5	2eC	.	.	AR{50}	.	.	{64}AV	.
6	.	.	.	.	.	.	.	.
7	4bC	.	.	FQ	.	.	.	.
8	5eC	{40}AN{57}	.	.	.	.	.	.
9	.	.	.	.	.	.	.	.
10	7eB	.	.	.	.	TY	.	.
11	.	.	.	.	.	.	.	.
12	9eB	.	KE	.	.	.	.	.
13	.	.	.	.	.	.	.	.
14	11eB	{30}VL	.	LE	.	.	.	.
15	.	.	.	.	.	.	.	.
16	13eB	SN{68}	.	.	.	.	.	.
17	14eB	.	.	.	.	.	.	{53}ED
18	.	.	.	.	.	.	.	.
19	.	.	.	.	.	.	.	.
20	17eH	DE	.	.	.	.	.	ED
21	18bH	ED{56}	.	{66}DG	.	.	{61}DE	DN
22	.	.	.	.	.	.	.	.
23	20bH	.	.	.	.	ML	.	.
24	21bH	.	.	.	.	{59}KR	.	.
25	22bH	.	.	.	.	.	.	AV
26	.	.	.	.	.	.	.	.
27	24eT	GD	.	.	.	.	.	.
28	.	.	.	.	.	.	.	.
29	.	.	.	.	.	.	.	.
30	25bC	VI	.	.	.	.	IV	.
31	26eC	{63}GD	.	.	.	DN	.	.
32	27eH	{57}LF	.	.	.	FV{68}	.	.
33	.	.	.	.	.	.	.	.
34	29bH	.	.	.	.	TL	.	.
35	.	.	.	.	.	.	.	.
36	.	.	.	.	.	.	.	.
37	32eH	.	.	.	.	.	.	IL
38	33bH	.	.	.	.	.	.	AV
39	34eH	{45}AV{28}	.	.	{28}VK{29}	{29}KN{33}	.	{26}LC
40	35eT	{44}AL{59}	59}LR{48}	.	.	.	.	.
41	.	.	.	.	.	.	.	.
42	37eC	.	.	KT	.	.	.	.
43	38bC	.	PQ	.	.	.	.	.
44	39eB	.	.	.	.	{32}TD	.	.
45	40bB	{50}VK	.	.	.	.	.	.
46	41eB	EV	.	VI	.	VE	.	.
47	.	.	.	.	.	.	.	.
48	43eB	{41}TV	.	.	.	.	.	{63}VI

49	.	.	.	.	.	.	.	.
50	45eB	.	.	.	{52}DN{51}	{51}ND{52}	.	.
51	.	.	.	.	.	.	.	.
52	.	.	.	.	.	.	.	.
53	.	.	.	.	.	.	.	.
54	.	.	.	.	.	.	.	.
55	.	.	.	.	.	.	.	.
56	.	.	.	.	.	.	.	.
57	.	.	.	.	.	.	.	.
58	48eB	{41}KN{48}	.	.	.	{44}NH	.	.
59	49bB	.	.	.	.	FM	.	.
60	50eB	.	TK{56}	.	.	.	TI	.
61	51bB	.	IT	.	.	.	.	.
62	52eB	.	.	.	.	{57}KR	.	.
63	.	.	.	.	.	.	.	.
64	54eB	{35}SN{41}	.	.	.	{40}NL	.	.
65	55bC	.	.	.	.	.	.	ST
66	.	.	.	.	.	.	.	.
67	57eT	.	.	.	.	.	.	FL
68	.	.	.	.	.	.	.	.
69	.	.	.	.	.	.	.	.
70	60eB	TY	.	.	.	.	.	.
71	61eB	.	.	ED	EI{34}	.	.	.
72	62bB	IV{69}	.	VL	.	VM{63}	.	.
73	63eB	{50}ND{52}	.	.	.	.	.	.
74	.	.	.	.	.	.	.	.
75	65eB	.	KT	.	.	KQ{43}	.	.
76	66bT	{40}IV	.	.	.	.	.	VL
77	.	.	.	.	.	.	.	.
78	68eB	.	.	EV	.	EK{44}	.	.
79	.	.	.	.	.	.	.	.
80	.	.	.	.	.	.	.	.
81	71eB	.	.	.	.	DE	.	.
82	.	.	.	.	.	.	.	.
83	73eB	ED	DH{39}	.	.	.	.	.
84	74bC	.	.	.	.	TL	.	.
85	75eT	{66}AK	.	.	.	.	{47}KT	{61}RG
86	76eT	.	.	.	.	.	.	{64}SP
87	77eT	.	.	.	.	LI{57}	.	.
88	.	.	.	.	.	.	.	.
89	79eC	GN	.	.	.	.	{50}ND	.
90	.	.	.	.	.	.	.	.
91	81eB	.	.	{36}KN{63}	.	.	.	.
92	82bB	.	CV	.	.	.	.	.
93	83eB	.	.	.	.	KQ{47}	{47}QM	.
94	84bB	ST{68}	.	.	.	.	.	.
95	85bB	.	.	.	.	LI{58}	{58}IT	.
96	.	.	.	.	.	.	.	.
97	87bB	.	.	.	.	.	TS	TQ
98	.	.	.	.	.	.	.	.
99	89eB	.	.	DE	.	.	.	{65}DN
100	90eT	.	.	.	.	.	.	.
101	91eT	.	.	.	.	.	.	.
102	92eB	.	.	KT	.	.	.	.
103	.	.	.	.	.	.	.	.

104	.	.	.	.	.	.	.	.
105	.	.	.	.	.	.	.	.
106	.	.	.	.	.	.	.	.
107	.	.	.	.	.	.	.	.
108	94bB	.	.	.	.	.	VQ	.
109	95bB	{27}KC	.	.	.	.	.	.
110	96eB	.	.	.	.	.	.	VE
111	.	.	.	.	.	.	.	.
112	98eB	.	.	.	.	.	.	KH
113	.	.	.	.	.	.	.	.
114	100eT	DE	.	.	.	CA	.	.
115	101bT	{42}DK	.	.	.	.	.	.
116	102eT	KE{55}	.	.	.	.	.	.
117	103eT	{28}EN	.	.	.	.	NG	.
118	104bC	TR	.	.	.	.	.	.
119	105bB	{40}TG	.	.	.	.	.	.
120	.	.	.	.	.	.	.	.
121	107eB	.	TK	.	.	.	.	.
122	108bB	RQ	.	.	.	.	.	.
123	.	.	.	.	.	.	.	.
124	110bB	.	.	IV	.	.	.	.
125	.	.	.	.	.	.	.	.
126	.	.	.	.	.	.	.	.
127	113eT	.	.	.	.	.	.	.
128	.	.	.	.	.	.	.	.
129	114eB	.	.	.		.	{47}KE	MF
130	.	.	.	.	.	.	.	.
131	116eB	{62}IH	.	HY	.	.	.	.
132	.	.	.	.	.	.	.	.
133	118eB	TE	.	.	.	.	.	EH
134	119bB	LM{59}	59}ML{59}	.	.	.	.	.
135	120eB	.	.	.	.	.	TR	TS
136	121bB	.	.	.	.	CA	.	.
137	122bT	{36}GE	.	{46}EG	.	.	.	.
138	123eT	.	.	.	.	.	DG	.
139	124eB	VQ	.	.	.	.	{34}QV	.
140	125eB	.	.	.	.	.	.	VT
141	.	.	.	.	.	.	.	.
142	127eB	TK	KR{67}	.	.	.	.	{56}KI
143	128bB	RQ	.	.	.	.	.	.
144	129bB	.	.	.	.	.	.	VI
145	130bB	YF	.	.	.	.	.	.
146	131eB	.	.	.	.	.	.	{68}KE
147	132eB	RK{56}	.	.	.	.	.	.
148	133eC	.	AK	.	.	AV	.	VI
149	.	{59}AN{44}	.	.	.	.	.	{51}RK
